# Genome-wide analyses reveal a highly conserved Dengue virus envelope peptide which is critical for virus viability and antigenic in humans

**DOI:** 10.1038/srep36339

**Published:** 2016-11-02

**Authors:** Renata C. Fleith, Francisco P. Lobo, Paula F. dos Santos, Mariana M. Rocha, Juliano Bordignon, Daisy M. Strottmann, Daniel O. Patricio, Wander R. Pavanelli, Maria Lo Sarzi, Claudia N. D. Santos, Brian J. Ferguson, Daniel S. Mansur

**Affiliations:** 1 Laboratory of Immunobiology, Department of Microbiology, Immunology and Parasitology, Universidade Federal de Santa Catarina, Florianópolis, Brazil; 2 Department of General Biology, Universidade Federal de Minas Gerais, Belo Horizonte, Brazil; 3 Laboratory of Molecular Virology, Instituto Carlos Chagas, Curitiba, Brazil; 4 Department of Pathology, Universidade Estadual de Londrina, Brazil; 5 Secretary for Health, Cambé, Brazil; 6 Department of Pathology, University of Cambridge, Cambridge, UK

## Abstract

Targeting regions of proteins that show a high degree of structural conservation has been proposed as a method of developing immunotherapies and vaccines that may bypass the wide genetic variability of RNA viruses. Despite several attempts, a vaccine that protects evenly against the four circulating *Dengue virus* (DV) serotypes remains elusive. To find critical conserved amino acids in dengue viruses, 120 complete genomes of each serotype were selected at random and used to calculate conservation scores for nucleotide and amino acid sequences. The identified peptide sequences were analysed for their structural conservation and localisation using crystallographic data. The longest, surface exposed, highly conserved peptide of Envelope protein was found to correspond to amino acid residues 250 to 270. Mutation of this peptide in DV1 was lethal, since no replication of the mutant virus was detected in human cells. Antibodies against this peptide were detected in DV naturally infected patients indicating its potential antigenicity. Hence, this study has identified a highly conserved, critical peptide in DV that is a target of antibodies in infected humans.

Dengue is a mosquito-borne disease caused by a group of viruses collectively known as *Dengue virus*, belonging to the *Flaviviridae* family. It affects nearly 390 million people every year worldwide^1^ with symptoms ranging from mild fever to severe shock syndrome. Although recently different vaccination strategies have achieved reasonable levels of protection, a vaccine that protects uniformly against the circulating serotypes is not available^2^. This is partly due to the high variability among these viruses, which can lead to partially protective immune responses and antibody dependent enhancement (ADE) of infection when non-neutralising antibodies facilitate virus entry by Fc gamma receptors^3^,^4^,^5^,^6^.

Dengue viruses (DV) are enveloped, ssRNA viruses with a single open reading frame encoding three structural and seven non-structural proteins^7^. The genetic variance amongst DV results in diverse immune responses such that they are classified into four serogroups (DV1–4) based on antigenic diversity^8^,^9^. Although mutations occur randomly in the genome, viral proteins show a combination of regions that are permissive to multiple mutations, which enable immune evasion through antigenic variation, and regions where amino acid residues critical for structure and viral fitness are conserved^10^. In general, regions exposed to the immune system are prone to variation, however, even the envelope (E) protein, the main antigenic determinant on the virion, retains highly conserved cryptic peptides^10^,^11^. It has been demonstrated that those conserved regions in structural proteins have an important role in viral fitness and might be targets of broadly neutralising antibodies in viruses such as HIV and Influenza A virus. For instance, although the majority of the protective immune response against influenza virus is provided by antibodies against the head of haemagglutinin (HA), new classes of multi-neutralising antibodies have been isolated that target the highly conserved HA stalk region^12^,^13^,^14^,^15^,^16^,^17^. Antibodies with similar properties have also been found that target functionally conserved regions of HIV glycoprotein 120^18^,^19^,^20^,^21^,^22^. In both cases these regions are being evaluated as vaccine targets and the antibodies elicited have been used to study immunoprophylaxis strategies.

In this context, the sequence conservation of DV was evaluated, with the aim of identifying conserved regions in the E protein. All complete genome sequences available on access date for DV4 in NCBI (120 sequences) were analysed along with the same number of genomes for each other DV serotype, that were randomly selected through their NCBI sorting numbers (access on November, 26, 2013). This unbiased dataset, comprising 480 sequences (all sequence IDs in Supplementary Data) allowed ample representation of the known variability observed in this taxonomic unit. The 480 GenBank files obtained in the previous step were processed using custom PERL scripts written using BioPerl module^23^ to extract protein and coding sequences. MUSCLE software was then used to align protein sequences with default parameters and these alignments, together with CDS data, were used to create a codon alignment. For both protein and codon alignments conservation scores were calculated based on the ratio of the count for the most frequent character (amino acids or nucleotides plus gaps) at a given position and the total number of sequences evaluated (480). To detect local conserved regions the fitting of a smooth curve to protein and codon conservation score data was carried out using smooth.spline function implemented in R language with smoothing parameter set to 0.4. The conservation scores varied from 0.4 to 1 (scores for all peptides are available in Supplementary Table), where a score closer to 1 corresponds to a higher conservation of the region across all analysed sequences (Fig. 1a). Although structural proteins are more variable in general, some regions in E demonstrated to be conserved as the non-structural proteins average. To analyse the E protein with greater resolution we repeated this procedure with its sequences separated from the full genome. This revealed two main peaks of conservation on the envelope protein (Fig. 1b). Further analyses revealed that the first of these sites is the fusion peptide. This peptide was first described in 1989, comprises a hydrophobic loop highly conserved in all flaviviruses, that is normally buried at the E dimeric form and becomes exposed at the tip of the fusogenic trimer (Fig. 2a,b)^24^,^25^,^26^. The second most significant peak of conservation was also mapped into domain II of the E protein and comprised residues 250–270 (polyprotein residues 530–551). Internally located in pre-fusion E dimer, covering the final residues of the ij loop, the αB helix and the beginning of the kl loop, the structure of this peptide (from now E_250–270_) was visualised using available structures of DV E proteins in pre- and post-fusion forms (Fig. 2a,b). E_250–270_ is the longest conserved peptide that is solvent-exposed in the E protein trimer, which is responsible for fusion of the viral envelope to the host membrane (Fig. 2b). Comparison of the localisation and structure of this DV peptide with those of other flavivirus E proteins shows its cross-species conservation (Fig. 2a,b). To visualise this conservation at the amino-acid sequence level, WebLogo was used^27^ to create a representation for the region spanning the peptide (columns 530–551). WebLogo was run using standard parameters and considering equiprobable amino acid frequencies. The same strategy was employed to compare the conservation of E_250–270_ in other flaviviruses. 120 sequences of *Japanese encephalitis virus* (JEV), 120 of *Tick-borne encephalitis virus* (TBEV), 120 of *West Nile virus* (WNV), were selected randomly from GenBank as well as the 70 sequences of *Yellow fever virus* (YFV) and 63 of *Zika virus* (ZKV), available, aligned with MUSCLE and visualized all together with WebLogo. Sixteen out of 21 amino acids in this peptide were 100% conserved in every DV sequence evaluated, being DV4 the most divergent among the four serotypes. When compared to other Flaviviruses at least seven amino acids from E_250–270_ are highly conserved (V250, L253, G254, Q256, G258, L264, G266; Fig. 2c and Supplementary Figure). These data indicate that E_250–270_ is conserved both at the sequence and structural levels across multiple flaviviruses.

As there is no specific reported function associated with the region where E_250–270_ is located in DV, to establish the significance of this highly conserved peptide, a DV was generated with a mutated E_250–270_ peptide (Fig. 3a). Several hydrophobic side chains were removed by mutation of five amino acid residues to alanine. These changes were designed to remove some of the most conserved side chains across all E protein sequences whilst minimising structural disruption across the peptide. Prediction of the effect of these substitutions on the three-dimensional structure of the envelope protein was carried out with Modeller^28^ using the complete E protein x-ray crystallographic structure as a template^29^ (Fig. 3b). PROCHECK^30^, WHAT IF^31^ and Verify3D^32^ algorithms were used to ensure the satisfaction of stereochemical restraints indicating that the amino acid substitutions could be tolerated by the E protein without gross structural disruption. More studies will be necessary to fully evaluate the impact of these mutations on E protein biology.

An infectious clone of DV1, strain BR/90, was used to construct the mutant virus^33^. Firstly, three silent nucleotide changes were inserted to add restriction enzyme cleavage sites (C368T, T1663G, G1822C, based on GenBank AF226685.2) and then alanine substitutions were introduced in E protein amino acids 250–253 and 255 by gene synthesis and conventional cloning (Fig. 3a). The resultant infectious clone, named Conserved Surface Mutant 1 (CSMut1), was fully sequenced and no modifications other than those desired were observed. CSMut1 and matching wild type (WT) were *in vitro* transcribed using MegaScript T7 synthesis kit (Ambion), supplemented with m^7^G(5′)ppp(5′)G RNA Cap Structure Analog (New England Biolabs). RNA was purified (RNeasy Mini Kit, Qiagen), and transfected into Huh7.5 cells with Lipofectin (Invitrogen). Cells and supernatant were recovered at indicated hours post transfection (h.p.t.) and the RNA extracted using RNeasy Mini Kit or QIAamp Viral RNA Mini Kit, respectively (both Qiagen). Using RT-qPCR for non-structural protein 5 (NS5) mRNA (5′ GCAAACATCTTCAGGGGAAGT 3′, 5′ GCTCCCGTACCTCTCCTACC 3′), only decreasing quantities of CSMut1 NS5 transcript were observed in both cell associated RNA or culture supernatant, suggesting an impaired ability of this mutant virus to replicate (Fig. 3c). The WT virus, on the other hand retained detectable levels of cell-associated DV RNA and increasing amounts of DV RNA in the culture supernatant consistent with efficient replication with the expected kinetics for this virus (Fig. 3c). Furthermore, CSMut1 growth was not detected by plaque assay (data not shown).

The impact of CSMut1 on DV1 was also assessed by immunofluorescence for the viral envelope antigen in Huh7.5 cells^34^. Briefly, Huh7.5 cells were transfected with wild type or CSMut1 RNA using Lipofectin, fixed after 120 hours, and stained with monoclonal anti-E antibody (4G2 - ATCC^®^ HB-112^™^), using Alexa Fluor 488 rabbit anti-mouse IgG (H + L, Life Technologies) as a secondary antibody, and DAPI counter stain (Molecular Probes). In agreement with RNA detection (Fig. 3c), E protein expression was not observed in cells transfected with CSMut1 RNA, suggesting that the mutant virus was not able to spread in Huh7.5 cells (Fig. 3d). To understand whether the impairment caused by CSMut1 was restricted to the mammalian host, similar experiments to those described above were performed in a C6/36 *Aedes albopictus* cell line. The results were remarkably similar to those in human cells (data not shown), suggesting that the defect caused by the mutations were not due to a host specific factor.

These data show that, despite the great diversity among the serotypes of dengue viruses there are at least two polypeptides within its main antigenic determinant that are highly conserved at both the sequence and structural levels. These two regions were also reported during analysis of pan-DV sequences as potential immune-relevant T cell determinants^11^. These peptides are generally buried in dimeric E form, and are thought to become fully exposed only in the E fusogenic form of mature virion^35^. This assumption suggests that once hidden to the humoral immune response, these regions remain conserved due to the absence of selective pressure from host immunity. To test this, the presence of antibodies against E_250–270_ was assessed in patient sera. Serum samples were obtained from an outbreak in Paraná state, Brazil (2013) according to the approved guidelines of Fiocruz (Fundação Oswaldo Cruz), within Instituto Carlos Chagas (Curitiba, Brazil). Experiments involving human subjects were approved by the committee of ethics and research (Comitê de Ética e Pesquisa – CEP) of Fiocruz-RJ under protocol 617/11. Informed consent was obtained from all donors. 70 serum samples were evaluated for DV infection (by NS1, IgM and IgG ELISAs). Samples corresponded to 54 DV1 sera designated positive by at least one assay and from patients in the acute phase of disease (up to the seventh day after the onset of symptoms) and 16 control sera from non-infected individuals. An indirect ELISA protocol was used to identify anti-E_250–270_ IgG antibodies^36^ (Fig. 4). Briefly, plates were coated with synthetic E_253–270_ peptide (it was not possible to solubilise a full length E_250–270_ peptide), blocked and incubated with serum samples. An HRP-linked, goat anti-human IgG (H + L, Invitrogen) was used as secondary antibody, and the plates were treated with o-phenylenediamine in citrate phosphate buffer containing 30% hydrogen peroxide. These ELISA data demonstrated this peptide to be antigenic in human natural infections, despite its predicted buried nature in the dimeric E form. One explanation for the development of antibodies to this site would be the dynamic movement of the virus particle, described as ‘breathing’^37^,^38^. In agreement, Ramanathan, B. *et al*.^39^ predict the peptide as being a potential linear epitope, and the Rey group described a broadly neutralising monoclonal antibody that interacts with the first valine of E_250–270_^40^. Therefore, two other reasons for conservation could be explored i) structure and ii) function. Modelling of the mutant E protein did not show evidence of structure destabilisation, even though the mutated residues were conserved across almost all analysed virus sequences, suggesting that E_250–270_ might have a functional role in the DV life cycle. In agreement with our data other groups suggest that amino acids within this region are key to the E protein biology. Accordingly, a single substitution at V251A moderately restricts DV replication and viral particle production in C6/36 and Vero cells, and reduces viral E protein detection by immunofluorescence in C6/36 and BHK-21 transfected cells^41^. Moreover, it was demonstrated that G266 and I270 substitutions to tryptophan affected viral replication in mammals and insect cells, and the I270W reduced fusion^42^. In contrast the study of Christian *et al*. using replicons with random point mutations in E protein did not verify significant differences in E expression, budding and infectivity when mutating residues of the peptide´s N-terminus (such as V251, and others mutated in CSMut1), or residues 266 and 270^43^. This could be related due to the amino acid utilized in substitutions, restrictions of the replicon´s method, and the contribution of multiple mutations as present in CSMut1. On the other hand, they suggests that other residues in peptide´s C-terminus, M258, H259 and A265, could have important role in fusion and, if mutated, greatly reduce or practically abolish viral replication^43^. These residues could form latch contacts with M proteins preventing the premature triggering of E protein. M258 forms with other residues a hydrophobic path that appears to be important to interact with F400 of E stem region in DV1 trimer, and histidine protonation, among them H259, enable the dissociation of E and M protein contacts^29^,^43^. The undetectable level of replication observed to CSMut1 demonstrated the importance of E_250–270_ to viral infectivity and further investigations should be done to elucidate the exact function this conserved peptide. Moreover the detection of antibodies anti E_250–270_ in natural infected patients point the possibility to the use of these peptide as immunological target, as recently shown^36^.

Our strategy enables identification of the most conserved regions in DV genomes, other flavivirus such as ZKV, and also the rational designing of mutant viruses to investigate the importance of these regions in viral fitness and infectivity. As carried out for E_250–270_ peptide, a systematic analysis of other highly conserved regions could suggest potential immunological/pharmacologic target for dengue treatment and control. Moreover, these knowledge can be extrapolated for other ssRNA genome viruses, and contribute to understand the evolution of their cryptic conserved peptides.

## Additional Information

**How to cite this article**: Fleith, R. C. *et al*. Genome-wide analyses reveal a highly conserved Dengue virus envelope peptide which is critical for virus viability and antigenic in humans. *Sci. Rep.* **6**, 36339; doi: 10.1038/srep36339 (2016).

**Publisher’s note:** Springer Nature remains neutral with regard to jurisdictional claims in published maps and institutional affiliations.

## Supplementary Material

Supplementary Information

## Figures and Tables

**Figure 1:**
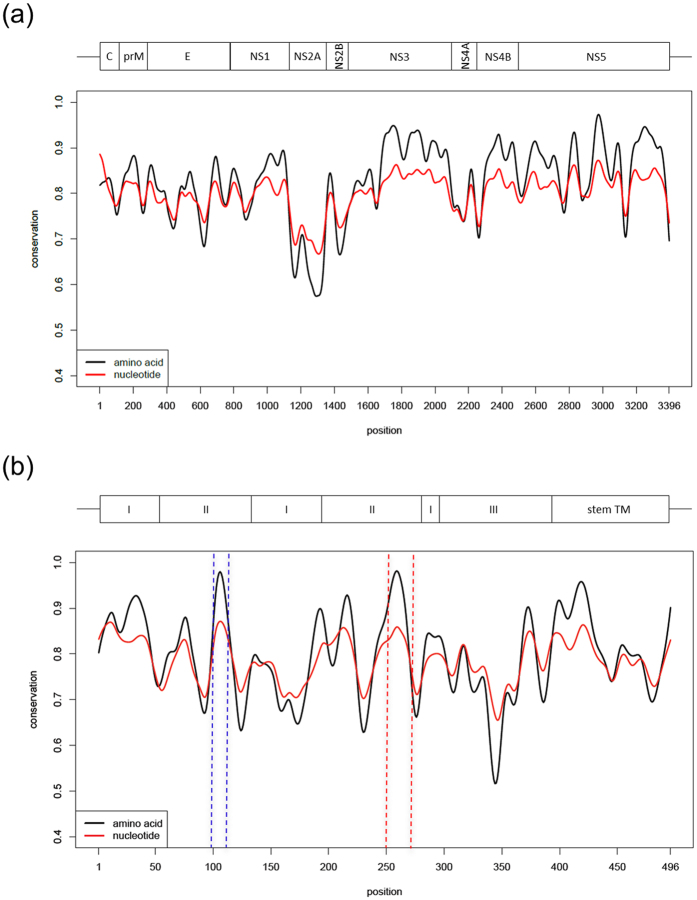
Genome-wide analysis of DV conserved sites. Conservation profile of nucleotides (red line) and amino acids (black line) of 480 dengue coding sequences (CDS) (**a**) and their respective envelope proteins (**b**). The “x” axis represents the position of amino acid residues in the DV CDS (**a**) or Envelope protein (**b**) and “y” axis represents the conservation score, where 1 indicates the highest conservation. The two most highly conserved peptides in the envelope protein (**b**) are the fusion peptide (residues E_99–112_, highlighted in the blue box) and E_250–270_ (red box). Above “a” the schematic DV CDS. C: capsid; prM: membrane precursor; E: envelope; NS: non-structural. Above “b” the scheme of E protein domains (I, II, II), the stem segment and transmembrane anchor (TM).

**Figure 2:**
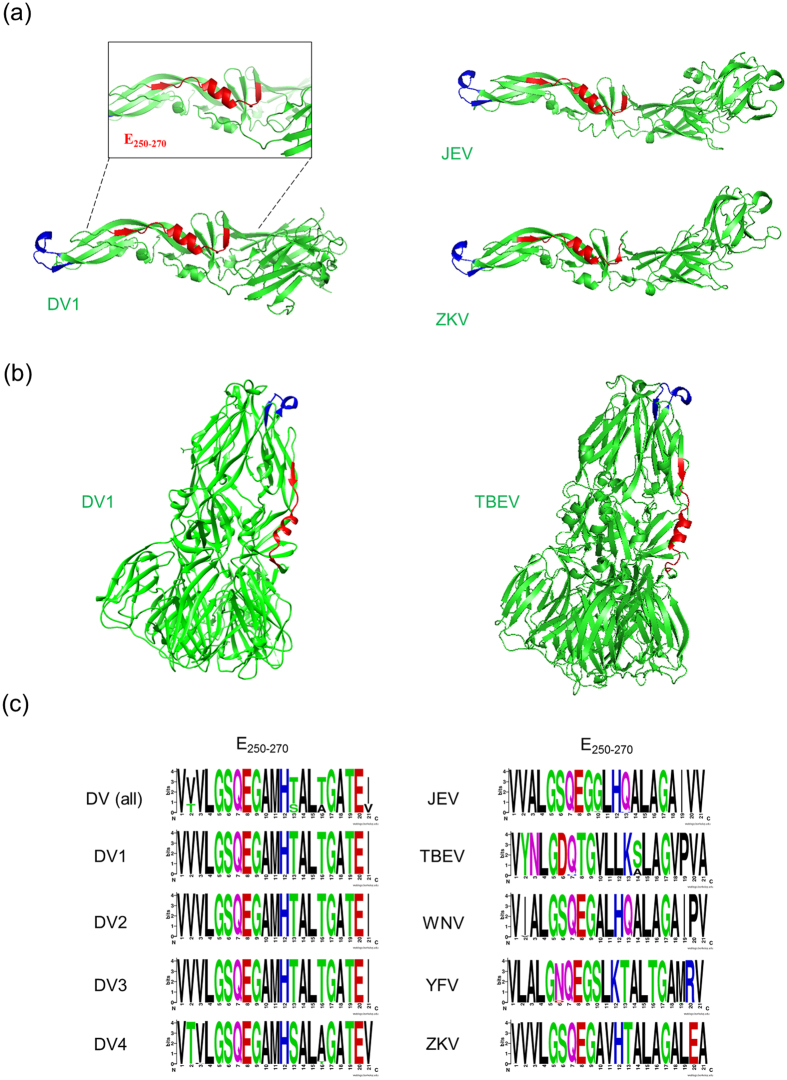
E_250–270_ sequence and structural conservation in different flaviviruses. (**a**) The fusion peptide (in blue) and E_250–270_ (in red) are highlighted in envelope monomer structures of DV1, JEV and ZKV and (**b**) in the trimeric form of DV1 and TBEV. PDB ids: 4GSX, 3P54, 5JHM and 1URZ. (**c**) WebLogo schematic showing the amino acid composition per site in E_250–270_ of DV and other flavivirus. Polar amino acids (G,S,T,Y,C) are colored in green, neutral (Q,N) in purple, basic (K,R,H) in blue, acidic (D,E) in red and hydrophobic (A,V,L,I,P,W,F,M) in black.

**Figure 3:**
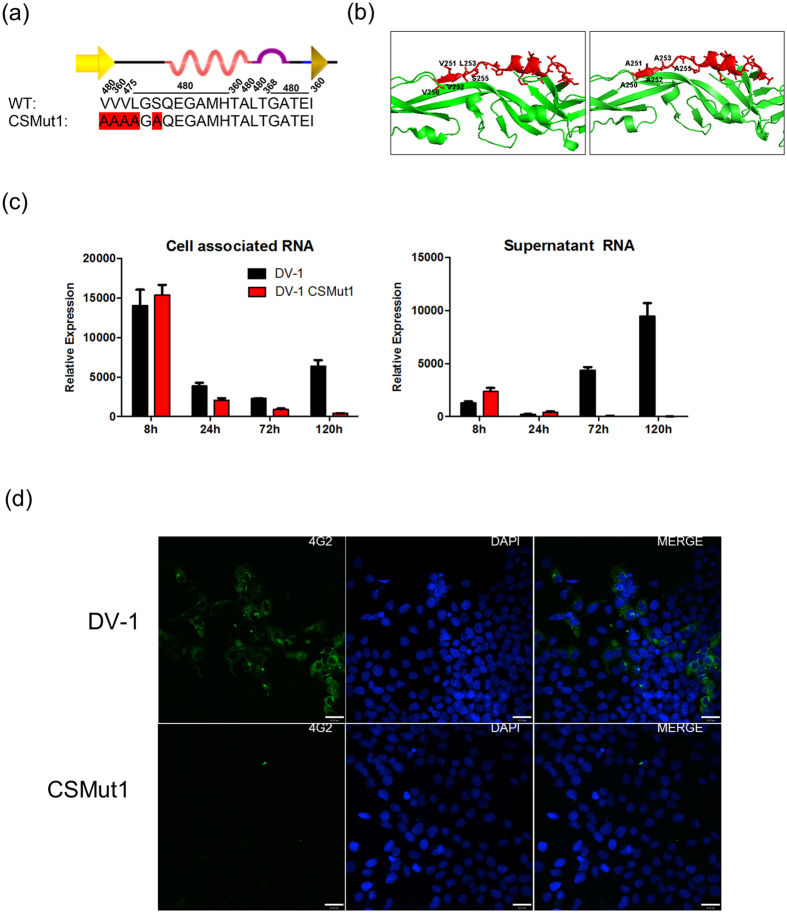
CSMut1 design, modelling and infectivity. (**a**) The wild type E_250–270_ and CSMut1 sequences are shown with mutated amino acids highlighted in red. (**b**) Wild type (on the left) and CSMut1 model (on the right) with E_250–270_ in red. (**c**) Relative quantification of DV NS5 RNA in Huh7.5 cells transfected with wild type and CSMut1 genomes at different time points. Cell associated RNA is shown in the left panel and supernatant extracted RNA in the right panel. (**d**) DV E-protein immunofluorescence staining of WT and CSMut1 120 hours after genome transfection in Huh7.5 cells (bars correspond to 32 μM).

**Figure 4:**
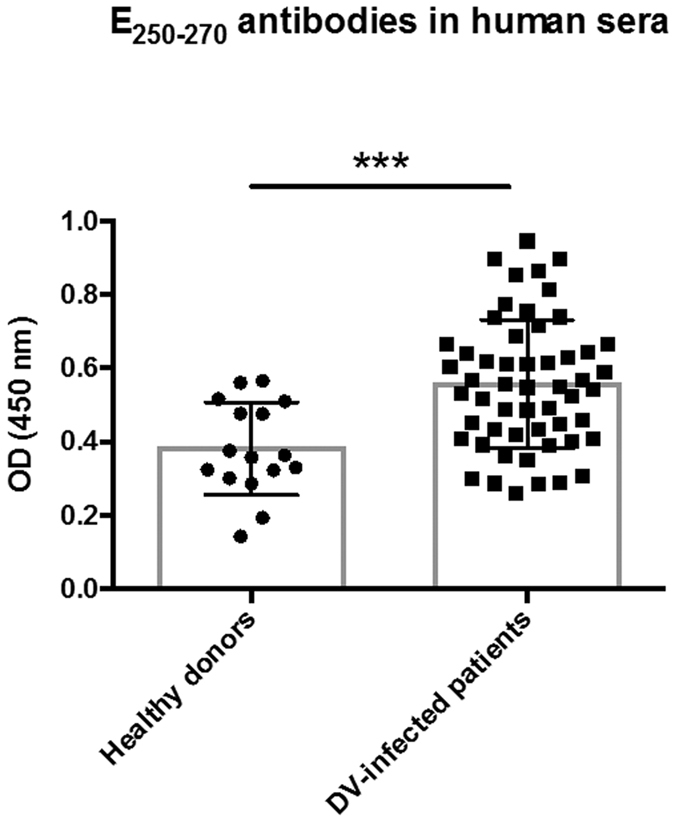
E_250–270_ antigenicity. Presence of antibodies against E_250–270_ analysed by ELISA. 54 serum samples positive for DV infection and 16 from non-infected controls. Absorbance values read at 450nm. ***p = 0.0004, error bars are ± standard deviation.

## References

[b1] Bhatt S. . The global distribution and burden of dengue. Nature 496, 504–507, doi: 10.1038/nature12060 (2013). 23563266 PMC3651993

[b2] Schwartz L. M., Halloran M. E., Durbin A. P. & Longini I. M. Jr. The dengue vaccine pipeline: Implications for the future of dengue control. Vaccine 33, 3293–3298, doi: 10.1016/j.vaccine.2015.05.010 (2015). 25989449 PMC4470297

[b3] Sabin A. B. Research on dengue during World War II. The American journal of tropical medicine and hygiene 1, 30–50 (1952). 14903434 10.4269/ajtmh.1952.1.30

[b4] Halstead S. B. & O’Rourke E. J. Antibody-enhanced dengue virus infection in primate leukocytes. Nature 265, 739–741 (1977). 404559 10.1038/265739a0

[b5] Dejnirattisai W. . Cross-reacting antibodies enhance dengue virus infection in humans. Science 328, 745–748, doi: 10.1126/science.1185181 (2010). 20448183 PMC3837288

[b6] Goncalvez A. P., Engle R. E. St., Claire M., Purcell R. H. & Lai C. J. Monoclonal antibody-mediated enhancement of dengue virus infection *in vitro* and *in vivo* and strategies for prevention. Proceedings of the National Academy of Sciences of the United States of America 104, 9422–9427, doi: 10.1073/pnas.0703498104 (2007). 17517625 PMC1868655

[b7] Chambers T. J., Hahn C. S., Galler R. & Rice C. M. Flavivirus genome organization, expression, and replication. Annual review of microbiology 44, 649–688, doi: 10.1146/annurev.mi.44.100190.003245 (1990). 2174669

[b8] Kuno G., Chang G. J., Tsuchiya K. R., Karabatsos N. & Cropp C. B. Phylogeny of the genus Flavivirus. Journal of virology 72, 73–83 (1998). 9420202 10.1128/jvi.72.1.73-83.1998 PMC109351

[b9] Holmes E. C. & Twiddy S. S. The origin, emergence and evolutionary genetics of dengue virus. Infection, genetics and evolution: journal of molecular epidemiology and evolutionary genetics in infectious diseases 3, 19–28 (2003). 10.1016/s1567-1348(03)00004-2 12797969

[b10] Drake J. W. & Holland J. J. Mutation rates among RNA viruses. Proceedings of the National Academy of Sciences of the United States of America 96, 13910–13913 (1999). 10570172 10.1073/pnas.96.24.13910 PMC24164

[b11] Khan A. M. . Conservation and variability of dengue virus proteins: implications for vaccine design. PLoS neglected tropical diseases 2, e272, doi: 10.1371/journal.pntd.0000272 (2008). 18698358 PMC2491585

[b12] Ekiert D. C. . A highly conserved neutralizing epitope on group 2 influenza A viruses. Science 333, 843–850, doi: 10.1126/science.1204839 (2011). 21737702 PMC3210727

[b13] Corti D. . A neutralizing antibody selected from plasma cells that binds to group 1 and group 2 influenza A hemagglutinins. Science 333, 850–856, doi: 10.1126/science.1205669 (2011). 21798894

[b14] Wang T. T. . Broadly protective monoclonal antibodies against H3 influenza viruses following sequential immunization with different hemagglutinins. PLoS pathogens 6, e1000796, doi: 10.1371/journal.ppat.1000796 (2010). 20195520 PMC2829068

[b15] Wang T. T. . Vaccination with a synthetic peptide from the influenza virus hemagglutinin provides protection against distinct viral subtypes. Proceedings of the National Academy of Sciences of the United States of America 107, 18979–18984, doi: 10.1073/pnas.1013387107 (2010). 20956293 PMC2973924

[b16] Sui J. . Structural and functional bases for broad-spectrum neutralization of avian and human influenza A viruses. Nature structural & molecular biology 16, 265–273, doi: 10.1038/nsmb.1566 (2009). PMC2692245 19234466

[b17] Tharakaraman K., Subramanian V., Cain D., Sasisekharan V. & Sasisekharan R. Broadly neutralizing influenza hemagglutinin stem-specific antibody CR8020 targets residues that are prone to escape due to host selection pressure. Cell host & microbe 15, 644–651, doi: 10.1016/j.chom.2014.04.009 (2014). 24832457 PMC4258880

[b18] Balazs A. B. . Antibody-based protection against HIV infection by vectored immunoprophylaxis. Nature 481, 81–84, doi: 10.1038/nature10660 (2012). PMC3253190 22139420

[b19] Walker L. M. . Broad neutralization coverage of HIV by multiple highly potent antibodies. Nature 477, 466–470, doi: 10.1038/nature10373 (2011). 21849977 PMC3393110

[b20] Wu X. . Rational design of envelope identifies broadly neutralizing human monoclonal antibodies to HIV-1. Science 329, 856–861, doi: 10.1126/science.1187659 (2010). 20616233 PMC2965066

[b21] Caskey M. . Viraemia suppressed in HIV-1-infected humans by broadly neutralizing antibody 3BNC117. Nature 522, 487–491, doi: 10.1038/nature14411 (2015). 25855300 PMC4890714

[b22] Scharf L. . Broadly Neutralizing Antibody 8ANC195 Recognizes Closed and Open States of HIV-1 Env. Cell 162, 1379–1390, doi: 10.1016/j.cell.2015.08.035 (2015). 26359989 PMC4587768

[b23] Stajich J. E. . The Bioperl toolkit: Perl modules for the life sciences. Genome research 12, 1611–1618, doi: 10.1101/gr.361602 (2002). 12368254 PMC187536

[b24] Roehrig J. T., Hunt A. R., Johnson A. J. & Hawkes R. A. Synthetic peptides derived from the deduced amino acid sequence of the E-glycoprotein of Murray Valley encephalitis virus elicit antiviral antibody. Virology 171, 49–60 (1989). 2472704 10.1016/0042-6822(89)90509-6

[b25] Roehrig J. T., Johnson A. J., Hunt A. R., Bolin R. A. & Chu M. C. Antibodies to dengue 2 virus E-glycoprotein synthetic peptides identify antigenic conformation. Virology 177, 668–675 (1990). 2371772 10.1016/0042-6822(90)90532-v

[b26] Seligman S. J. Constancy and diversity in the flavivirus fusion peptide. Virology journal 5, 27, doi: 10.1186/1743-422X-5-27 (2008). 18275613 PMC2275255

[b27] Crooks G. E., Hon G., Chandonia J. M. & Brenner S. E. WebLogo: a sequence logo generator. Genome research 14, 1188–1190, doi: 10.1101/gr.849004 (2004). 15173120 PMC419797

[b28] Marti-Renom M. A. . Comparative protein structure modeling of genes and genomes. Annual review of biophysics and biomolecular structure 29, 291–325, doi: 10.1146/annurev.biophys.29.1.291 (2000). 10940251

[b29] Klein D. E., Choi J. L. & Harrison S. C. Structure of a dengue virus envelope protein late-stage fusion intermediate. Journal of virology 87, 2287–2293, doi: 10.1128/JVI.02957-12 (2013). 23236058 PMC3571469

[b30] Laskowski R. A., Moss D. S. & Thornton J. M. Main-chain bond lengths and bond angles in protein structures. Journal of molecular biology 231, 1049–1067, doi: 10.1006/jmbi.1993.1351 (1993). 8515464

[b31] Vriend G. WHAT IF: a molecular modeling and drug design program. Journal of molecular graphics 8, 52-56, 29 (1990). 10.1016/0263-7855(90)80070-v 2268628

[b32] Luthy R., Bowie J. U. & Eisenberg D. Assessment of protein models with three-dimensional profiles. Nature 356, 83–85, doi: 10.1038/356083a0 (1992). 1538787

[b33] Suzuki R., de Borba L., Duarte dos Santos C. N. & Mason P. W. Construction of an infectious cDNA clone for a Brazilian prototype strain of dengue virus type 1: characterization of a temperature-sensitive mutation in NS1. Virology 362, 374–383, doi: 10.1016/j.virol.2006.11.026 (2007). 17289102 PMC2396755

[b34] Zanluca C., Mazzarotto G. A., Bordignon J. & Duarte Dos Santos C. N. Development, characterization and application of monoclonal antibodies against Brazilian Dengue virus isolates. PloS one 9, e110620, doi: 10.1371/journal.pone.0110620 (2014). 25412181 PMC4239016

[b35] Modis Y., Ogata S., Clements D. & Harrison S. C. Structure of the dengue virus envelope protein after membrane fusion. Nature 427, 313–319, doi: 10.1038/nature02165 (2004). 14737159

[b36] Mareze V. A. . Tests in mice of a dengue vaccine candidate made of chimeric Junin virus-like particles and conserved dengue virus envelope sequences. Applied microbiology and biotechnology 100, 125–133, doi: 10.1007/s00253-015-6973-7 (2016). 26386688

[b37] Cockburn J. J. . Mechanism of dengue virus broad cross-neutralization by a monoclonal antibody. Structure 20, 303–314, doi: 10.1016/j.str.2012.01.001 (2012). 22285214

[b38] Lok S. M. . Binding of a neutralizing antibody to dengue virus alters the arrangement of surface glycoproteins. Nature structural & molecular biology 15, 312–317, doi: 10.1038/nsmb.1382 (2008). 18264114

[b39] Ramanathan B. . Synthetic B-Cell Epitopes Eliciting Cross-Neutralizing Antibodies: Strategies for Future Dengue Vaccine. PloS one 11, e0155900, doi: 10.1371/journal.pone.0155900 (2016). 27223692 PMC4880327

[b40] Rouvinski A. . Recognition determinants of broadly neutralizing human antibodies against dengue viruses. Nature 520, 109–113, doi: 10.1038/nature14130 (2015). 25581790

[b41] Kroschewski H., Sagripanti J. L. & Davidson A. D. Identification of amino acids in the dengue virus type 2 envelope glycoprotein critical to virus infectivity. The Journal of general virology 90, 2457–2461, doi: 10.1099/vir.0.011486-0 (2009). 19587132

[b42] Butrapet S. . Amino acid changes within the E protein hinge region that affect dengue virus type 2 infectivity and fusion. Virology 413, 118–127, doi: 10.1016/j.virol.2011.01.030 (2011). 21353281

[b43] Christian E. A. . Atomic-level functional model of dengue virus Envelope protein infectivity. Proceedings of the National Academy of Sciences of the United States of America 110, 18662–18667, doi: 10.1073/pnas.1310962110 (2013). 24158478 PMC3831943

