# Supplementary material for: Genome-wide analyses reveal a highly conserved Dengue virus envelope peptide which is critical for virus viability and antigenic in humans
Source: Sci Rep. 2016 Nov 2;6:36339. doi: 10.1038/srep36339 (PMC5090869; doi:10.1038/srep36339)
Supplement: Supplementary Information [file srep36339-s1.pdf]

Scientific Reports

Genome-wide analyses reveal a highly conserved Dengue virus envelope peptide which is critical for virus viability and antigenic in humans

Renata C. Fleith, Francisco P. Lobo, Paula F. Santos, Mariana M. Rocha, Juliano Bordignon, Daisy M. Strottmann, Daniel O. Patricio, Wander R. Pavanelli, Maria Lo-Sarzi, Claudia N. D. Santos, Brian J. Ferguson, Daniel S. Mansur

Supplementary Data:

List of used sequence IDs

Dengue virus sequence Ids

AB189121.1
AB189122.1
AB189126.1
AB189128.1
AB214879.1
AF022440.1
AF119661.1
AF169683.1
AF226685.2
AF298807.1
AF326573.1
AF489932.1
AY145121.1
AY496874.2
AY618988.1
AY618989.1
AY618990.1
AY618991.1
AY618992.1
AY618993.1
AY676353.1
AY702040.1
AY732478.1
AY732482.1
AY762085.1
AY835999.4
AY858036.2
AY858037.2
AY858042.2
AY876494.1
AY923865.1
AY947539.1

DQ181803.1
DQ448231.2
DQ672561.1
DQ675522.1
DQ675525.1
EF032590.1
EF457905.1
EF457906.1
EU081181.1
EU081183.1
EU081185.1
EU081190.1
EU081192.1
EU081193.1
EU081203.1
EU081204.1
EU081211.1
EU081212.1
EU081215.1
EU081220.1
EU081223.1
EU081231.1
EU081232.1
EU081260.1
EU081267.1
EU482455.1
EU482457.1
EU482460.1
EU482461.1
EU482464.1
EU482466.1
EU482482.1
EU482490.1
EU482492.1
EU482502.1
EU482530.1
EU482535.1
EU482539.1
EU482540.1
EU482547.1
EU482551.1
EU482554.1
EU482574.1
EU482580.1
EU482599.1
EU482618.1
EU482621.1
EU482623.1
EU482642.1
EU482653.1
EU482654.1
EU482659.1
EU482661.1
EU482677.1
EU482683.1
EU482699.1

EU482700.1
EU482708.1
EU482710.1
EU482711.1
EU482727.1
EU482734.1
EU482753.1
EU482755.1
EU482757.1
EU482769.1
EU482786.1
EU482794.1
EU482800.1
EU482803.1
EU482827.1
EU529693.1
EU529694.1
EU529699.1
EU569690.1
EU569695.1
EU569706.1
EU569708.1
EU569717.1
EU596493.1
EU660395.1
EU660400.1
EU660420.1
EU677147.1
EU677154.1
EU677160.1
EU677174.1
EU687220.1
EU687222.1
EU687230.1
EU687232.1
EU687233.1
EU726772.1
EU726773.1
EU726779.1
EU854295.1
EU854296.1
EU854297.1
EU854299.1
EU854300.1
EU854301.1
FJ024424.1
FJ024436.1
FJ024451.1
FJ024458.1
FJ024476.1
FJ182008.1
FJ182016.1
FJ182017.1
FJ182024.1
FJ182030.1
FJ182037.1

FJ226067.1
FJ373302.1
FJ390375.1
FJ410174.1
FJ410194.1
FJ410202.1
FJ410205.1
FJ410211.1
FJ410219.1
FJ410230.1
FJ410231.1
FJ410237.1
FJ410250.1
FJ410258.1
FJ410269.1
FJ410272.1
FJ410274.1
FJ410285.1
FJ410288.1
FJ432721.1
FJ432726.1
FJ432728.1
FJ432731.1
FJ432736.1
FJ432739.1
FJ432742.1
FJ432743.1
FJ461311.1
FJ461316.1
FJ461334.1
FJ461339.1
FJ469909.1
FJ547061.1
FJ547072.1
FJ547073.1
FJ547075.1
FJ547077.1
FJ547082.1
FJ562103.1
FJ639693.1
FJ639695.1
FJ639697.1
FJ639698.1
FJ639712.1
FJ639715.1
FJ639716.1
FJ639721.1
FJ639726.1
FJ639736.1
FJ639737.1
FJ639738.1
FJ639739.1
FJ639742.1
FJ639744.1
FJ639745.1
FJ639748.1

FJ639751.1
FJ639757.1
FJ639762.1
FJ639764.1
FJ639769.1
FJ639771.1
FJ639773.1
FJ639781.1
FJ639795.1
FJ639802.1
FJ639805.1
FJ639816.1
FJ639826.1
FJ639830.1
FJ639834.1
FJ644564.1
FJ687437.1
FJ687439.1
FJ744700.1
FJ744725.1
FJ744728.1
FJ744731.1
FJ744738.1
FJ744741.1
FJ744745.1
FJ810409.1
FJ810417.1
FJ850057.1
FJ850058.1
FJ850059.1
FJ850065.1
FJ850082.1
FJ850094.1
FJ850095.1
FJ850098.1
FJ850099.1
FJ850103.1
FJ873808.1
FJ882535.1
FJ882538.1
FJ882548.1
FJ882557.1
FJ882560.1
FJ882578.1
FJ882580.1
FJ882581.1
FJ882582.1
FJ882583.1
FJ882584.1
FJ882585.1
FJ882586.1
FJ882587.1
FJ882588.1
FJ882589.1
FJ882590.1
FJ882591.1

FJ882592.1
FJ882595.1
FJ882596.1
FJ882597.1
FJ882598.1
FJ882599.1
FJ882600.1
FJ882601.1
FJ898384.1
FJ898386.1
FJ898392.1
FJ898433.1
FJ898436.1
FJ898451.1
FJ898470.1
FJ898473.1
FJ898479.1
FJ906963.1
FJ906967.1
FM210205.1
FM210207.1
FM210217.1
FM210221.1
FM210227.1
FM210233.2
FM210240.2
FM210242.2
FM210245.2
GQ199773.1
GQ199802.1
GQ199824.1
GQ199834.1
GQ199862.1
GQ199865.1
GQ199874.1
GQ199876.1
GQ199878.1
GQ199879.1
GQ199880.1
GQ199881.1
GQ199882.1
GQ199883.1
GQ199884.1
GQ199885.1
GQ199891.1
GQ252675.1
GQ398256.1
GQ398265.1
GQ398268.1
GQ398270.1
GQ398281.1
GQ398288.1
GQ398299.1
GQ398307.1
GQ868498.1
GQ868500.1

GQ868505.1
GQ868528.1
GQ868532.1
GQ868539.1
GQ868551.1
GQ868554.1
GQ868563.1
GQ868574.1
GQ868579.1
GQ868580.1
GQ868581.1
GQ868582.1
GQ868583.1
GQ868584.1
GQ868585.1
GQ868590.1
GQ868591.1
GQ868594.1
GQ868598.1
GQ868617.1
GQ868628.1
GQ868630.1
GQ868631.1
GQ868636.1
GQ868642.1
GQ868643.1
GQ868644.1
GQ868645.1
GU131693.1
GU131696.1
GU131727.1
GU131735.1
GU131757.1
GU131764.1
GU131772.1
GU131777.1
GU131780.1
GU131788.1
GU131794.1
GU131814.1
GU131836.1
GU131841.1
GU131846.1
GU131848.1
GU131855.1
GU131856.1
GU131857.1
GU131865.1
GU131874.1
GU131883.1
GU131896.1
GU131898.1
GU131901.1
GU131902.1
GU131906.1
GU131912.1

GU131920.1
GU131932.1
GU131936.1
GU131948.1
GU131967.1
GU131968.1
GU131970.1
GU289913.1
GU363549.1
HM181934.1
HM181954.1
HM181958.1
HM181971.1
HM181974.1
HM181977.1
HM181978.1
HM582099.1
HM582102.1
HM582107.1
HM582109.1
HM582112.1
HM582115.1
HM631859.1
HM756276.1
HM756277.1
HQ166031.1
HQ332172.1
HQ332173.1
HQ332174.1
HQ332175.1
HQ332176.1
HQ332181.1
HQ541786.1
HQ541795.1
HQ541798.1
HQ705610.1
HQ705612.1
HQ705622.1
HQ705623.1
HQ733861.1
JF262779.1
JF262780.1
JF262781.1
JF262782.1
JF262783.1
JF295012.1
JF459993.1
JF730044.1
JF730054.1
JF730055.1
JF741967.1
JF920402.1
JF920406.1
JF937604.1
JF937605.1
JF937615.1

JF937624.1
JF937637.1
JF937651.1
JF937652.1
JN000937.1
JN406514.1
JN559740.2
JN559741.2
JN638338.1
JN638341.1
JN638570.1
JN638571.1
JN638572.1
JN697058.1
JN697379.1
JN819403.1
JN819406.1
JN819408.1
JN819409.1
JN983813.1
JQ045688.1
JQ513330.1
JQ513331.1
JQ513332.1
JQ513333.1
JQ513334.1
JQ513335.1
JQ513336.1
JQ513337.1
JQ513338.1
JQ513339.1
JQ513340.1
JQ513341.1
JQ513342.1
JQ513343.1
JQ513344.1
JQ513345.1
JQ822247.1
JX024757.1
JX024758.1
JX079690.1
JX669469.1
JX669477.1
JX669483.1
JX669486.1
JX669487.1
JX669497.1
JX669500.1
JX669504.1
KC333651.1
KC692517
KF954945
NC_001474.2
NC_001475.2
NC_001477.1
NC_002640.1

Tick-borne encephalitis virus sequence IDs

AB062063.2
AB062064.1
AB753012.1
AF069066.1
AY169390.3
DQ401140.3
DQ862460.1
DQ989336.1
EF469661.1
EF469662.1
EU816450.1
EU816451.1
EU816452.1
EU816453.1
EU816454.1
EU816455.2
FJ402885.1
FJ402886.1
FJ572210.1
FJ906622.1
FJ968751.1
FJ997899.1
GQ228395.1
GQ266392.1
GU121642.1
GU183379.1
GU183381.1
GU183382.1
GU183384.1
HM120875.1
HM535610.1
HM535611.1
HM859894.1
HM859895.1
HQ201303.1
HQ901366.1
HQ901367.1
JF316707.1
JF316708.1
JF819648.2
JN003205.1
JN003206.1
JN003207.1
JN003208.1
JN003209.1
JN229223.1
JQ650522.1
JQ650523.1
JQ825144.1
JQ825145.1
JQ825147.1

JQ825148.1
JQ825149.1
JQ825150.1
JQ825152.1
JQ825154.1
JQ825155.1
JQ825156.1
JQ825158.1
JQ825159.1
JQ825160.1
JQ825163.1
JQ825164.1
JX498939.1
JX498940.1
JX534167.1
KC414090.1
KC422663.2
KC422667.2
KC806252.1
KC835595.1
KC835596.1
KC835597.1
KF151173.1
KF826915.1
KF826916.1
KF880803.1
KF880804.1
KF880805.1
KF951037.1
KF991107.1
KJ000002.1
KJ626343.1
KJ633033.1
KJ701416.1
KJ739729.1
KJ739730.1
KJ739731.1
KJ755186.1
KJ914682.1
KJ914683.1
KJ922512.1
KJ922513.1
KJ922514.1
KJ922515.1
KJ922516.1
KM019545.1
KM019546.1
KP331441.1
KP331442.1
KP331443.1
KP345889.1
KP644245.1
KP844724.1
KP844725.1
KP844726.1
KP844727.1

KP869172.1
KP938507.1
KT001070.1
KT001071.1
KT001072.1
KT001073.1
KT224352.1
KT224353.1
LC017691.1
LC017692.1
LC017693.1
NC_001672.1
U27495.1

West Nile virus sequence IDs

KJ501450.1
AF404754.1
AF404755.1
AY712946.1
AY712947.1
DQ164191.1
DQ164199.1
DQ164206.1
DQ377179.1
DQ411029.1
DQ666448.1
EF429200.1
EF530047.1
FJ151394.1
HM051416.1
HM488127.1
HM488130.1
HM488134.1
HM488147.1
HM488151.1
HM488154.1
HM488186.1
HM488187.1
HM488189.1
HM488191.1
HM488193.1
HM488201.1
HM488212.1
HM488217.1
HM488219.1
HM488228.1
HM488231.1
HM488234.1
HM488237.1
HM488244.1
HM488248.1
HM488249.1
HM756656.1

HM756657.1
HM756658.1
HM756671.1
HQ671670.1
HQ671675.1
HQ671689.1
HQ671695.1
HQ671701.1
HQ671723.1
HQ671726.1
HQ705673.1
HQ891012.1
JF415930.1
JF719066.1
JF920306.1
JF920728.1
JF920732.1
JF920736.1
JF920737.1
JF920739.1
JF920745.1
JF920749.1
JF957164.1
JF957179.1
JF957184.1
JF957185.1
JF972636.1
JN819320.1
JN819323.1
JX041631.1
JX041632.1
JX123031.1
JX556213.1
KC736489.1
KC736499.1
KC736500.1
KF647248.1
KJ501108.1
KJ501114.1
KJ501123.1
KJ501126.1
KJ501131.1
KJ501133.1
KJ501135.1
KJ501146.1
KJ501170.1
KJ501214.1
KJ501229.1
KJ501257.1
KJ501258.1
KJ501277.1
KJ501290.1
KJ501299.1
KJ501300.1
KJ501302.1
KJ501303.1

KJ501305.1
KJ501306.1
KJ501309.1
KJ501321.1
KJ501339.1
KJ501346.1
KJ501355.1
KJ501376.1
KJ501393.1
KJ501411.1
KJ501422.1
KJ501428.1
KJ501443.1
KJ501451.1
KJ501460.1
KJ501463.1
KJ501464.1
KJ501477.1
KJ501480.1
KJ501484.1
KJ501495.1
KJ501511.1
KJ501520.1
KJ501526.1
KJ786936.1
M12294.2

Japanese encephalitis virus

AY303791.1
FJ495189.1
KT957423.1
L48961.1
AF221499.1
KT957420.1
KC196115.1
JX131374.1
GQ902063.1
GQ902062.1
GQ902060.1
GQ902059.1
GU187972.1
AF045551.2
KT957422.1
KR265316.1
JX050179.1
AY303796.1
AY303793.1
AY303792.1
EF623988.1
EF623987.1
GU556217.1
EU693899.1
AY585242.1

EU880214.1
AF315119.1
EF623989.1
EF107523.1
KT229574.1
KF297916.1
KF297915.1
AY303798.1
AY303795.1
JQ086763.1
JF499790.1
JF499789.1
HQ893545.1
JF706285.1
JF706281.1
JF706282.1
JF706283.1
JF706284.1
JF706280.1
JF706279.1
JF706278.1
JF706277.1
JF706276.1
JF706275.1
JF706274.1
JF706273.1
JF706272.1
JF706270.1
JF706269.1
JF706268.1
JF706267.1
JN381873.1
JN381872.1
JN381871.1
JN381870.1
JN381869.1
JN381868.1
JN381867.1
JN381866.1
JN381865.1
JN381864.1
JN381863.1
JN381861.1
JN381862.1
JN381860.1
JN381859.1
JN381858.1
JN381857.1
JN381856.1
JN381855.1
JN381854.1
JN381853.1
JN381851.1
JN381852.1
JN381850.1
JN381849.1

JN381847.1
JN381848.1
JN381846.1
JN381844.1
JN381845.1
JN381843.1
JN381842.1
JN381839.1
JN381840.1
JN381841.1
JN381838.1
JN381837.1
JN381836.1
JN381835.1
JN381834.1
JN381833.1
JN381832.1
JF915894.1
JN381830.1
JN381831.1
HM596272.1
AF098735.1
AF098737.1
AF069076.1
AY316157.1
U47032.1
GQ918133.2
JN711458.1
JN711459.1
JQ031753.1
KC915016.1
KF907505.1
KM677246.1
AF254452.1
AF254453.1
FJ185036.1
EU429297.1
GU205163.1
EF543861.1

Yellow fever virus sequence IDs

AF052437.1
AF052438.1
AF052439.1
AF052444.1
AF052445.1
AF052446.1
AF094612.1
AY572535.1
AY603338.1
AY640589.1
AY968064.1
AY968065.1

DQ100292.1
DQ118157.1
DQ235229.1
FJ654700.1
GQ379162.1
GQ379163.1
HM582851.1
JF912179.1
JF912180.1
JF912181.1
JF912182.1
JF912183.1
JF912184.1
JF912185.1
JF912186.1
JF912187.1
JF912188.1
JF912189.1
JF912190.1
JN620362.1
JN628279.1
JN628280.1
JN628281.1
JN811140.1
JN811141.1
JN811142.1
JN811143.1
JX503529.1
JX898868.1
JX898869.1
JX898870.1
JX898871.1
JX898872.1
JX898873.1
JX898874.1
JX898875.1
JX898876.1
JX898877.1
JX898878.1
JX898879.1
JX898880.1
JX898881.1
JX949181.1
KF769015.1
KF769016.1
KF907504.1
KM388814.1
KM388815.1
KM388816.1
KM388817.1
KM388818.1
NC_002031.1
U17066.1
U17067.1
U21055.1
U21056.1

U54798.1
X03700.1

Zika virus

KU955595.1
KU955594.1
KU955593.1
KU955592.1
KU955591.1
KX056898.1
KU866423.1
KU681081.3
KU681082.3
KU321639.1
KX117076.1
KU509998.3
KU870645.1
KU926310.1
KU926309.1
KU922960.1
KU922923.1
KU820898.1
KU744693.1
KU853012.1
KU853013.1
KU740184.2
KU497555.1
KU707826.1
KU527068.1
KU501215.1
KU647676.1
LC002520.1
KJ776791.1
KX087102.1
KX051563.1
KX087101.1
KU963796.1
KU991811.1
KU820899.2
KU729217.2
KU720415.1
KU761564.1
KU729218.1
KU365779.1
NC_012532.1
KU501217.1
KU501216.1
KU365780.1
KU365778.1
KU365777.1
KU312312.1
KF383119.1
KF383118.1

EU545988.1
AY632535.2
KF383115.1
KF383116.1
KF383117.1
KU955590.1
KU955589.1
KU963574.1
KU963573.1
KU820897.1
KF268950.1
KF268949.1
KF268948.1
DQ859059.1

Supplementary Table:

Mean conservation profile for all peptides of length 20, computed for the 480 DENV aligned polyproteins.

To generate this Supplementary Table was used a sliding window algorithm to compute mean conservation profile values for all peptides of length 20 across the alignment of the 480 DENV polyproteins, and sorted peptides based on mean conservation scores.

START	END	AVERAGE_CONSERVATION_PROFILE
2837	2857	0.9999
2835	2855	0.9999
2836	2856	0.9999
2362	2382	0.9998
2366	2386	0.9880
2367	2387	0.9880
2368	2388	0.9880
2371	2391	0.9880
2369	2389	0.9880
2370	2390	0.9880
2365	2385	0.9880
2372	2392	0.9880
2364	2384	0.9879
2363	2383	0.9879
2374	2394	0.9877
2373	2393	0.9877
2375	2395	0.9877
2376	2396	0.9876
2457	2477	0.9875
2458	2478	0.9875
2838	2858	0.9874
2945	2965	0.9874
2944	2964	0.9874
2943	2963	0.9874
2961	2981	0.9873
2951	2971	0.9873
2952	2972	0.9873
2947	2967	0.9873
2956	2976	0.9873
2948	2968	0.9873
2962	2982	0.9873
2459	2479	0.9873
2965	2985	0.9873
2946	2966	0.9873
2953	2973	0.9873
2954	2974	0.9873
2949	2969	0.9873
2950	2970	0.9873
2963	2983	0.9873
2964	2984	0.9873

2460	2480	0.9872
2955	2975	0.9872
2461	2481	0.9872
1727	1747	0.9772
1726	1746	0.9772
1728	1748	0.9772
2968	2988	0.9749
2971	2991	0.9749
1963	1983	0.9749
2969	2989	0.9749
1962	1982	0.9749
2967	2987	0.9749
2972	2992	0.9749
2970	2990	0.9749
2966	2986	0.9749
2958	2978	0.9748
2833	2853	0.9748
2834	2854	0.9748
2957	2977	0.9748
2361	2381	0.9748
2960	2980	0.9748
2959	2979	0.9748
2463	2483	0.9747
2462	2482	0.9747
2464	2484	0.9746
2832	2852	0.9746
2831	2851	0.9746
1692	1712	0.9745
1834	1854	0.9745
1833	1853	0.9744
1832	1852	0.9744
3286	3306	0.9741
3285	3305	0.9741
3284	3304	0.9741
3231	3251	0.9736
3232	3252	0.9736
2808	2828	0.9714
2830	2850	0.9698
2829	2849	0.9698
2977	2997	0.9696
1760	1780	0.9657
1761	1781	0.9657
1759	1779	0.9657
1729	1749	0.9647
1725	1745	0.9644
3083	3103	0.9641
3082	3102	0.9641
3084	3104	0.9641
3085	3105	0.9641
2973	2993	0.9630
2974	2994	0.9629
529	549	0.9628
2377	2397	0.9626
3332	3352	0.9625
2456	2476	0.9625
2454	2474	0.9625

2455	2475	0.9625
2942	2962	0.9624
1961	1981	0.9624
1771	1791	0.9624
1960	1980	0.9624
1776	1796	0.9623
3333	3353	0.9623
1773	1793	0.9623
1958	1978	0.9623
3336	3356	0.9623
1774	1794	0.9623
1775	1795	0.9623
3335	3355	0.9623
1772	1792	0.9623
1777	1797	0.9623
1959	1979	0.9623
1957	1977	0.9623
3334	3354	0.9623
1685	1705	0.9623
3260	3280	0.9622
3222	3242	0.9622
3220	3240	0.9622
3221	3241	0.9622
3259	3279	0.9622
2003	2023	0.9622
3224	3244	0.9621
3223	3243	0.9621
1837	1857	0.9620
1835	1855	0.9620
1897	1917	0.9620
1836	1856	0.9620
1898	1918	0.9620
1684	1704	0.9618
3176	3196	0.9618
1907	1927	0.9617
1900	1920	0.9617
1901	1921	0.9617
1902	1922	0.9617
1899	1919	0.9617
1903	1923	0.9617
1906	1926	0.9617
1683	1703	0.9614
1735	1755	0.9612
1731	1751	0.9612
1732	1752	0.9612
1736	1756	0.9612
3226	3246	0.9612
3230	3250	0.9612
1733	1753	0.9612
3229	3249	0.9612
1734	1754	0.9612
3227	3247	0.9612
3228	3248	0.9612
3237	3257	0.9611
3236	3256	0.9611
3238	3258	0.9611

1851	1871	0.9604
1850	1870	0.9604
1847	1867	0.9604
1848	1868	0.9604
1846	1866	0.9603
1845	1865	0.9603
1844	1864	0.9603
2807	2827	0.9592
2975	2995	0.9571
2976	2996	0.9571
2987	3007	0.9569
2986	3006	0.9569
3251	3271	0.9558
3249	3269	0.9557
3245	3265	0.9557
3248	3268	0.9557
3250	3270	0.9557
3247	3267	0.9557
3246	3266	0.9557
3283	3303	0.9556
3282	3302	0.9555
1724	1744	0.9545
2359	2379	0.9540
2360	2380	0.9539
1758	1778	0.9531
1883	1903	0.9523
1757	1777	0.9523
1884	1904	0.9523
1885	1905	0.9523
1882	1902	0.9523
1750	1770	0.9523
1881	1901	0.9522
2839	2859	0.9522
1876	1896	0.9519
3080	3100	0.9519
3150	3170	0.9519
3149	3169	0.9519
3081	3101	0.9519
2993	3013	0.9514
1730	1750	0.9510
1966	1986	0.9507
530	550	0.9503
531	551	0.9502
1964	1984	0.9500
1691	1711	0.9499
2940	2960	0.9499
1689	1709	0.9499
1690	1710	0.9499
1688	1708	0.9499
3219	3239	0.9499
1770	1790	0.9499
2939	2959	0.9498
2938	2958	0.9498
1687	1707	0.9498
1769	1789	0.9498
1686	1706	0.9498

2000	2020	0.9497
2001	2021	0.9497
1955	1975	0.9497
1954	1974	0.9497
2002	2022	0.9497
2465	2485	0.9496
1694	1714	0.9496
1693	1713	0.9496
2467	2487	0.9496
2695	2715	0.9495
2696	2716	0.9495
2597	2617	0.9494
2598	2618	0.9494
2208	2228	0.9493
2207	2227	0.9493
3175	3195	0.9493
2212	2232	0.9493
2209	2229	0.9493
1099	1119	0.9492
1905	1925	0.9492
2205	2225	0.9492
2206	2226	0.9492
2211	2231	0.9492
1904	1924	0.9492
3090	3110	0.9490
2335	2355	0.9490
1681	1701	0.9489
1682	1702	0.9489
1680	1700	0.9489
3091	3111	0.9487
1737	1757	0.9487
3225	3245	0.9486
3235	3255	0.9486
3234	3254	0.9486
3233	3253	0.9486
2596	2616	0.9484
2595	2615	0.9484
999	1019	0.9481
2590	2610	0.9481
1738	1758	0.9480
1849	1869	0.9479
1748	1768	0.9479
1744	1764	0.9479
1746	1766	0.9479
1747	1767	0.9479
1745	1765	0.9479
1838	1858	0.9478
1839	1859	0.9478
3024	3044	0.9476
1840	1860	0.9474
1842	1862	0.9474
1841	1861	0.9474
1843	1863	0.9474
1000	1020	0.9474
2810	2830	0.9462
2809	2829	0.9462

2056	2076	0.9459
2055	2075	0.9459
3023	3043	0.9457
2694	2714	0.9456
2006	2026	0.9450
2008	2028	0.9450
2007	2027	0.9450
2828	2848	0.9447
2980	3000	0.9445
2978	2998	0.9445
2979	2999	0.9445
2822	2842	0.9444
2982	3002	0.9444
2823	2843	0.9444
2983	3003	0.9444
2827	2847	0.9444
2984	3004	0.9444
2821	2841	0.9444
2985	3005	0.9444
2824	2844	0.9444
2820	2840	0.9444
2981	3001	0.9444
2826	2846	0.9444
2825	2845	0.9444
3331	3351	0.9440
2691	2711	0.9434
2693	2713	0.9434
2692	2712	0.9434
3255	3275	0.9433
3254	3274	0.9433
3253	3273	0.9433
3258	3278	0.9432
3257	3277	0.9432
3256	3276	0.9432
3290	3310	0.9430
3293	3313	0.9430
3294	3314	0.9430
3289	3309	0.9429
3291	3311	0.9429
3287	3307	0.9429
3292	3312	0.9429
3288	3308	0.9429
3243	3263	0.9423
3244	3264	0.9423
3242	3262	0.9422
3241	3261	0.9422
3240	3260	0.9422
3239	3259	0.9420
1723	1743	0.9420
1722	1742	0.9419
2811	2831	0.9416
2812	2832	0.9416
2814	2834	0.9414
2813	2833	0.9414
1763	1783	0.9407
1765	1785	0.9407

1764	1784	0.9407
1762	1782	0.9407
1766	1786	0.9406
1767	1787	0.9406
1887	1907	0.9398
1751	1771	0.9398
1888	1908	0.9398
1756	1776	0.9398
1889	1909	0.9398
2272	2292	0.9398
1754	1774	0.9398
1890	1910	0.9398
1752	1772	0.9398
1886	1906	0.9398
1755	1775	0.9398
1753	1773	0.9398
1878	1898	0.9397
1879	1899	0.9397
1880	1900	0.9397
1891	1911	0.9396
1877	1897	0.9396
3151	3171	0.9396
2988	3008	0.9395
2992	3012	0.9395
2990	3010	0.9395
2989	3009	0.9395
2991	3011	0.9395
3148	3168	0.9394
2994	3014	0.9390
1749	1769	0.9388
3328	3348	0.9385
3329	3349	0.9384
1965	1985	0.9382
687	707	0.9381
686	706	0.9381
1968	1988	0.9380
992	1012	0.9380
1917	1937	0.9380
1969	1989	0.9380
1970	1990	0.9380
1915	1935	0.9380
1971	1991	0.9380
994	1014	0.9380
1967	1987	0.9380
1916	1936	0.9380
1972	1992	0.9380
993	1013	0.9380
528	548	0.9378
527	547	0.9378
1778	1798	0.9378
2378	2398	0.9376
2380	2400	0.9376
2381	2401	0.9376
1701	1721	0.9375
1698	1718	0.9375
2379	2399	0.9375

2449	2469	0.9375
377	397	0.9375
2448	2468	0.9375
2451	2471	0.9375
2450	2470	0.9375
1700	1720	0.9375
1699	1719	0.9375
2453	2473	0.9375
1697	1717	0.9375
376	396	0.9375
2452	2472	0.9375
2941	2961	0.9374
2629	2649	0.9373
3338	3358	0.9373
3337	3357	0.9373
3339	3359	0.9373
3261	3281	0.9373
684	704	0.9373
1997	2017	0.9372
1998	2018	0.9372
2004	2024	0.9372
1956	1976	0.9372
1996	2016	0.9372
1999	2019	0.9372
1695	1715	0.9371
1895	1915	0.9371
1696	1716	0.9371
2466	2486	0.9371
2273	2293	0.9371
1896	1916	0.9371
2204	2224	0.9370
1829	1849	0.9370
1831	1851	0.9370
524	544	0.9370
523	543	0.9370
1828	1848	0.9370
2198	2218	0.9369
2600	2620	0.9369
2599	2619	0.9369
2203	2223	0.9369
1994	2014	0.9368
2210	2230	0.9368
2336	2356	0.9368
3177	3197	0.9367
1026	1046	0.9365
1679	1699	0.9364
3146	3166	0.9364
3147	3167	0.9364
1995	2015	0.9364
1027	1047	0.9361
2280	2300	0.9361
1028	1048	0.9361
2279	2299	0.9361
1029	1049	0.9361
2213	2233	0.9359
2587	2607	0.9358

2592	2612	0.9355
3021	3041	0.9355
3020	3040	0.9355
1854	1874	0.9354
1852	1872	0.9354
1741	1761	0.9354
2593	2613	0.9354
1739	1759	0.9354
1853	1873	0.9354
2594	2614	0.9354
1743	1763	0.9354
1742	1762	0.9354
1740	1760	0.9354
3025	3045	0.9351
1872	1892	0.9344
1874	1894	0.9344
1873	1893	0.9344
3093	3113	0.9334
3092	3112	0.9334
2995	3015	0.9334
3094	3114	0.9334
2840	2860	0.9330
3330	3350	0.9330
3173	3193	0.9329
1086	1106	0.9328
1092	1112	0.9328
1088	1108	0.9328
1091	1111	0.9328
1085	1105	0.9328
1090	1110	0.9328
1087	1107	0.9328
1089	1109	0.9328
2009	2029	0.9326
1855	1875	0.9326
3298	3318	0.9318
2634	2654	0.9317
2635	2655	0.9317
2631	2651	0.9315
2383	2403	0.9310
3281	3301	0.9308
3275	3295	0.9308
3252	3272	0.9308
2690	2710	0.9308
3274	3294	0.9308
3273	3293	0.9308
3295	3315	0.9305
2998	3018	0.9298
928	948	0.9296
2818	2838	0.9292
2819	2839	0.9292
2815	2835	0.9291
2816	2836	0.9291
2817	2837	0.9291
2358	2378	0.9290
3099	3119	0.9288
3098	3118	0.9284

3095	3115	0.9283
3096	3116	0.9283
3097	3117	0.9283
1768	1788	0.9281
2044	2064	0.9280
958	978	0.9278
959	979	0.9278
957	977	0.9278
3152	3172	0.9276
1892	1912	0.9269
1875	1895	0.9269
1894	1914	0.9269
1893	1913	0.9269
3086	3106	0.9266
3087	3107	0.9265
3088	3108	0.9264
3089	3109	0.9264
3327	3347	0.9260
2276	2296	0.9258
2277	2297	0.9258
786	806	0.9256
787	807	0.9256
2566	2586	0.9255
688	708	0.9255
1913	1933	0.9254
1912	1932	0.9254
2568	2588	0.9254
1914	1934	0.9254
2569	2589	0.9253
1920	1940	0.9253
526	546	0.9253
1919	1939	0.9253
525	545	0.9253
790	810	0.9251
1921	1941	0.9251
788	808	0.9251
789	809	0.9251
379	399	0.9250
1918	1938	0.9250
378	398	0.9250
1908	1928	0.9250
3213	3233	0.9249
3215	3235	0.9249
2333	2353	0.9249
3214	3234	0.9249
2332	2352	0.9249
2572	2592	0.9248
2574	2594	0.9248
2330	2350	0.9248
2005	2025	0.9248
2573	2593	0.9248
1952	1972	0.9247
1953	1973	0.9247
2937	2957	0.9247
2564	2584	0.9246
2468	2488	0.9246

1826	1846	0.9246
1830	1850	0.9245
2698	2718	0.9245
211	231	0.9245
690	710	0.9245
691	711	0.9245
1827	1847	0.9245
2697	2717	0.9245
210	230	0.9245
2202	2222	0.9244
3079	3099	0.9244
2199	2219	0.9244
3179	3199	0.9242
3174	3194	0.9242
1100	1120	0.9242
1101	1121	0.9242
3178	3198	0.9242
2334	2354	0.9241
1678	1698	0.9237
2284	2304	0.9236
2281	2301	0.9236
2283	2303	0.9236
2282	2302	0.9236
173	193	0.9234
174	194	0.9234
2214	2234	0.9234
2586	2606	0.9233
2589	2609	0.9232
2588	2608	0.9232
2591	2611	0.9231
2806	2826	0.9217
1001	1021	0.9216
3145	3165	0.9213
2058	2078	0.9210
2996	3016	0.9209
2054	2074	0.9209
2057	2077	0.9209
3022	3042	0.9207
2061	2081	0.9207
3057	3077	0.9206
2841	2861	0.9205
3172	3192	0.9204
923	943	0.9203
922	942	0.9203
1084	1104	0.9203
2010	2030	0.9201
2011	2031	0.9201
1093	1113	0.9200
2012	2032	0.9200
1098	1118	0.9197
3300	3320	0.9195
3299	3319	0.9194
3296	3316	0.9193
2382	2402	0.9193
3297	3317	0.9193
2636	2656	0.9192

2633	2653	0.9192
2637	2657	0.9192
3198	3218	0.9190
2632	2652	0.9190
2630	2650	0.9190
2702	2722	0.9189
2703	2723	0.9189
1032	1052	0.9187
1031	1051	0.9187
2639	2659	0.9186
2638	2658	0.9186
3272	3292	0.9184
3276	3296	0.9184
3277	3297	0.9184
2045	2065	0.9178
2048	2068	0.9178
2047	2067	0.9178
2046	2066	0.9178
2050	2070	0.9178
2049	2069	0.9178
1717	1737	0.9177
1720	1740	0.9176
1718	1738	0.9176
1716	1736	0.9176
1715	1735	0.9176
1721	1741	0.9176
1719	1739	0.9176
2065	2085	0.9175
147	167	0.9173
148	168	0.9173
927	947	0.9171
989	1009	0.9168
991	1011	0.9164
990	1010	0.9164
3190	3210	0.9160
3189	3209	0.9160
3188	3208	0.9160
2357	2377	0.9158
956	976	0.9156
3154	3174	0.9151
3153	3173	0.9151
2071	2091	0.9144
302	322	0.9141
303	323	0.9141
304	324	0.9141
300	320	0.9141
305	325	0.9141
299	319	0.9141
301	321	0.9141
532	552	0.9140
995	1015	0.9136
996	1016	0.9136
200	220	0.9135
2278	2298	0.9134
2274	2294	0.9133
2275	2295	0.9133

1702	1722	0.9132
1703	1723	0.9131
2565	2585	0.9130
685	705	0.9130
1973	1993	0.9130
2567	2587	0.9130
2571	2591	0.9129
3340	3360	0.9128
2570	2590	0.9128
1911	1931	0.9127
998	1018	0.9126
997	1017	0.9126
2447	2467	0.9125
204	224	0.9125
1909	1929	0.9125
203	223	0.9125
1910	1930	0.9125
3218	3238	0.9124
3216	3236	0.9124
205	225	0.9124
3217	3237	0.9124
206	226	0.9124
682	702	0.9123
2331	2351	0.9123
3262	3282	0.9123
683	703	0.9123
3263	3283	0.9122
3078	3098	0.9122
1922	1942	0.9121
197	217	0.9121
2704	2724	0.9121
689	709	0.9120
522	542	0.9120
2337	2357	0.9120
2201	2221	0.9119
2197	2217	0.9119
1024	1044	0.9119
2200	2220	0.9119
1025	1045	0.9119
1801	1821	0.9119
1023	1043	0.9119
1539	1559	0.9119
2575	2595	0.9118
2576	2596	0.9118
2784	2804	0.9118
1993	2013	0.9118
680	700	0.9117
212	232	0.9117
3180	3200	0.9117
681	701	0.9117
1664	1684	0.9116
1985	2005	0.9115
1667	1687	0.9115
675	695	0.9114
1802	1822	0.9114
2603	2623	0.9114

676	696	0.9114
1668	1688	0.9114
674	694	0.9114
2602	2622	0.9114
2601	2621	0.9111
1030	1050	0.9111
2309	2329	0.9110
1665	1685	0.9110
1666	1686	0.9110
2585	2605	0.9109
673	693	0.9109
2563	2583	0.9108
2053	2073	0.9105
2052	2072	0.9105
2051	2071	0.9105
2446	2466	0.9103
3019	3039	0.9103
2445	2465	0.9102
2444	2464	0.9102
2443	2463	0.9101
2887	2907	0.9099
2433	2453	0.9099
2442	2462	0.9098
2440	2460	0.9098
2441	2461	0.9098
3061	3081	0.9097
2427	2447	0.9097
1860	1880	0.9095
1870	1890	0.9094
1869	1889	0.9094
3017	3037	0.9094
1871	1891	0.9094
2805	2825	0.9092
1609	1629	0.9091
1858	1878	0.9091
1859	1879	0.9091
1607	1627	0.9091
1610	1630	0.9091
1612	1632	0.9091
1608	1628	0.9091
1611	1631	0.9091
1102	1122	0.9090
1103	1123	0.9090
3341	3361	0.9086
3342	3362	0.9086
3343	3363	0.9086
1541	1561	0.9085
2059	2079	0.9084
2060	2080	0.9084
2064	2084	0.9083
2063	2083	0.9083
2062	2082	0.9083
3058	3078	0.9082
1825	1845	0.9079
924	944	0.9078
1856	1876	0.9076

3304	3324	0.9075
3303	3323	0.9073
3302	3322	0.9073
2043	2063	0.9071
2042	2062	0.9071
2069	2089	0.9071
2070	2090	0.9071
2040	2060	0.9070
2039	2059	0.9070
2271	2291	0.9070
2038	2058	0.9070
3301	3321	0.9070
925	945	0.9067
3212	3232	0.9066
3197	3217	0.9065
2700	2720	0.9065
3026	3046	0.9065
2701	2721	0.9065
3279	3299	0.9060
1033	1053	0.9060
2384	2404	0.9060
2699	2719	0.9059
3280	3300	0.9059
3278	3298	0.9059
2640	2660	0.9057
2997	3017	0.9056
145	165	0.9055
1714	1734	0.9051
1713	1733	0.9051
2067	2087	0.9050
2066	2086	0.9050
2068	2088	0.9050
146	166	0.9048
2999	3019	0.9048
3102	3122	0.9040
3101	3121	0.9038
3100	3120	0.9038
3187	3207	0.9035
3183	3203	0.9035
785	805	0.9035
3184	3204	0.9035
3191	3211	0.9034
3186	3206	0.9034
3185	3205	0.9034
953	973	0.9032
954	974	0.9031
955	975	0.9031
2072	2092	0.9019
2074	2094	0.9019
2073	2093	0.9019
533	553	0.9015
201	221	0.9006
1779	1799	0.9005
375	395	0.9005
288	308	0.9003
295	315	0.9002

791	811	0.9001
1623	1643	0.9000
1071	1091	0.9000
1072	1092	0.9000
1068	1088	0.9000
1069	1089	0.9000
1070	1090	0.9000
207	227	0.8999
2792	2812	0.8999
2794	2814	0.8999
208	228	0.8999
2793	2813	0.8999
1951	1971	0.8999
3077	3097	0.8999
3076	3096	0.8999
209	229	0.8999
199	219	0.8998
3075	3095	0.8998
2328	2348	0.8998
2934	2954	0.8998
2327	2347	0.8998
2326	2346	0.8998
2935	2955	0.8998
2329	2349	0.8998
2628	2648	0.8998
1977	1997	0.8997
1984	2004	0.8997
2936	2956	0.8997
1978	1998	0.8997
1979	1999	0.8997
1976	1996	0.8997
2624	2644	0.8997
2625	2645	0.8997
2796	2816	0.8996
1619	1639	0.8996
198	218	0.8996
2795	2815	0.8996
693	713	0.8996
2705	2725	0.8996
2797	2817	0.8996
694	714	0.8996
692	712	0.8996
2706	2726	0.8995
519	539	0.8995
521	541	0.8995
520	540	0.8995
2425	2445	0.8995
2799	2819	0.8995
2707	2727	0.8995
2798	2818	0.8995
518	538	0.8995
2426	2446	0.8994
2196	2216	0.8994
1538	1558	0.8994
1540	1560	0.8994
2785	2805	0.8994

1022	1042	0.8994
1676	1696	0.8993
1674	1694	0.8993
1675	1695	0.8993
678	698	0.8992
1988	2008	0.8992
1991	2011	0.8992
1987	2007	0.8992
679	699	0.8992
2215	2235	0.8992
517	537	0.8992
3182	3202	0.8992
677	697	0.8992
3181	3201	0.8992
1989	2009	0.8992
1990	2010	0.8992
213	233	0.8992
1671	1691	0.8989
2580	2600	0.8989
2579	2599	0.8989
2581	2601	0.8989
2577	2597	0.8989
2338	2358	0.8989
1669	1689	0.8989
1595	1615	0.8989
2578	2598	0.8989
1670	1690	0.8989
1677	1697	0.8987
1804	1824	0.8986
2285	2305	0.8986
1803	1823	0.8986
3060	3080	0.8985
2583	2603	0.8985
2584	2604	0.8985
2582	2602	0.8985
175	195	0.8984
176	196	0.8984
2325	2345	0.8984
2561	2581	0.8983
2559	2579	0.8983
2560	2580	0.8983
672	692	0.8983
2562	2582	0.8983
1345	1365	0.8982
2320	2340	0.8981
2321	2341	0.8981
3018	3038	0.8978
988	1008	0.8977
50	70	0.8977
2432	2452	0.8974
2431	2451	0.8974
2429	2449	0.8974
2430	2450	0.8974
3064	3084	0.8974
3065	3085	0.8974
2439	2459	0.8973

3063	3083	0.8973
3062	3082	0.8973
2436	2456	0.8972
1861	1881	0.8970
1867	1887	0.8969
1863	1883	0.8969
2884	2904	0.8969
1865	1885	0.8969
2885	2905	0.8969
1866	1886	0.8969
1864	1884	0.8969
1862	1882	0.8969
1868	1888	0.8969
1606	1626	0.8966
1603	1623	0.8966
1005	1025	0.8966
1003	1023	0.8966
1605	1625	0.8966
1004	1024	0.8966
1604	1624	0.8966
1002	1022	0.8965
1040	1060	0.8959
3059	3079	0.8957
3166	3186	0.8955
3169	3189	0.8954
3167	3187	0.8954
3170	3190	0.8954
3168	3188	0.8954
1083	1103	0.8953
2843	2863	0.8953
2844	2864	0.8953
2842	2862	0.8953
149	169	0.8952
3171	3191	0.8952
1095	1115	0.8950
1857	1877	0.8950
1094	1114	0.8950
3305	3325	0.8949
1346	1366	0.8947
3027	3047	0.8947
2041	2061	0.8947
1348	1368	0.8947
1096	1116	0.8947
1347	1367	0.8947
1097	1117	0.8947
926	946	0.8944
3205	3225	0.8941
3211	3231	0.8941
3016	3036	0.8941
3201	3221	0.8940
3203	3223	0.8940
3202	3222	0.8940
3204	3224	0.8940
2882	2902	0.8936
3264	3284	0.8935
3265	3285	0.8935

3269	3289	0.8935
3268	3288	0.8935
3266	3286	0.8935
3267	3287	0.8935
2689	2709	0.8934
3270	3290	0.8934
2688	2708	0.8934
3271	3291	0.8934
3028	3048	0.8933
2641	2661	0.8932
2643	2663	0.8930
2646	2666	0.8930
2642	2662	0.8930
1596	1616	0.8921
929	949	0.8921
2719	2739	0.8918
287	307	0.8916
2356	2376	0.8909
3192	3212	0.8909
784	804	0.8909
309	329	0.8906
1710	1730	0.8904
1709	1729	0.8904
960	980	0.8903
3155	3175	0.8900
2077	2097	0.8896
2076	2096	0.8895
172	192	0.8895
2075	2095	0.8895
298	318	0.8894
3326	3346	0.8885
1704	1724	0.8881
202	222	0.8881
371	391	0.8880
1974	1994	0.8880
1975	1995	0.8880
296	316	0.8877
49	69	0.8876
46	66	0.8876
48	68	0.8876
1076	1096	0.8875
1078	1098	0.8875
381	401	0.8875
1073	1093	0.8875
1077	1097	0.8875
1075	1095	0.8875
1074	1094	0.8875
380	400	0.8875
2791	2811	0.8874
1622	1642	0.8874
1364	1384	0.8874
2469	2489	0.8874
2626	2646	0.8873
3073	3093	0.8873
3074	3094	0.8873
1621	1641	0.8872

826	846	0.8872
1980	2000	0.8872
1982	2002	0.8872
1981	2001	0.8872
1620	1640	0.8871
1663	1683	0.8870
3306	3326	0.8870
2708	2728	0.8869
1537	1557	0.8869
1800	1820	0.8869
2786	2806	0.8869
1992	2012	0.8868
196	216	0.8868
827	847	0.8868
2783	2803	0.8868
830	850	0.8868
831	851	0.8868
829	849	0.8868
189	209	0.8867
516	536	0.8867
187	207	0.8866
188	208	0.8866
2606	2626	0.8865
2607	2627	0.8865
1986	2006	0.8865
2605	2625	0.8865
2604	2624	0.8864
2216	2236	0.8864
2217	2237	0.8864
1929	1949	0.8861
2310	2330	0.8860
2308	2328	0.8859
2307	2327	0.8859
671	691	0.8858
2312	2332	0.8858
2314	2334	0.8858
670	690	0.8858
2718	2738	0.8857
2557	2577	0.8857
2558	2578	0.8857
2313	2333	0.8857
178	198	0.8856
2319	2339	0.8855
987	1007	0.8854
179	199	0.8854
186	206	0.8853
483	503	0.8851
484	504	0.8850
51	71	0.8850
3069	3089	0.8849
2435	2455	0.8849
2434	2454	0.8849
2428	2448	0.8849
3067	3087	0.8849
3068	3088	0.8849
485	505	0.8849

3066	3086	0.8849
2886	2906	0.8849
1786	1806	0.8849
1785	1805	0.8848
1784	1804	0.8848
1780	1800	0.8848
1782	1802	0.8848
2888	2908	0.8848
1783	1803	0.8848
669	689	0.8847
14	34	0.8847
13	33	0.8847
1598	1618	0.8846
2547	2567	0.8845
1602	1622	0.8842
1344	1364	0.8842
2802	2822	0.8842
2803	2823	0.8842
2801	2821	0.8842
2804	2824	0.8842
2800	2820	0.8842
1039	1059	0.8841
1613	1633	0.8841
3103	3123	0.8841
1104	1124	0.8841
1823	1843	0.8840
1343	1363	0.8840
1105	1125	0.8839
3144	3164	0.8837
3346	3366	0.8837
3347	3367	0.8837
2546	2566	0.8836
2545	2565	0.8836
3345	3365	0.8835
3344	3364	0.8835
3056	3076	0.8833
3351	3371	0.8829
1082	1102	0.8828
921	941	0.8828
1810	1830	0.8826
2014	2034	0.8825
2013	2033	0.8825
1349	1369	0.8822
1353	1373	0.8822
1350	1370	0.8822
1351	1371	0.8822
3364	3384	0.8819
2270	2290	0.8819
3015	3035	0.8817
2399	2419	0.8816
2400	2420	0.8816
3210	3230	0.8815
3207	3227	0.8815
3206	3226	0.8815
3200	3220	0.8815
2398	2418	0.8815

3199	3219	0.8815
3209	3229	0.8815
3208	3228	0.8815
3196	3216	0.8814
3195	3215	0.8814
3194	3214	0.8814
486	506	0.8812
2687	2707	0.8809
3325	3345	0.8808
3307	3327	0.8806
143	163	0.8805
2645	2665	0.8805
2644	2664	0.8805
144	164	0.8804
1711	1731	0.8802
1712	1732	0.8802
1661	1681	0.8800
1662	1682	0.8800
1041	1061	0.8799
3001	3021	0.8797
480	500	0.8796
1597	1617	0.8796
3000	3020	0.8796
1038	1058	0.8794
1036	1056	0.8794
1037	1057	0.8794
2035	2055	0.8793
2037	2057	0.8793
2036	2056	0.8793
286	306	0.8791
276	296	0.8785
277	297	0.8785
783	803	0.8785
782	802	0.8785
2355	2375	0.8784
2354	2374	0.8784
2403	2423	0.8783
1705	1725	0.8782
2402	2422	0.8782
307	327	0.8781
308	328	0.8781
306	326	0.8781
310	330	0.8781
274	294	0.8780
1707	1727	0.8779
1706	1726	0.8779
1708	1728	0.8779
961	981	0.8778
2492	2512	0.8778
962	982	0.8777
2353	2373	0.8776
3159	3179	0.8775
3161	3181	0.8775
3157	3177	0.8775
3156	3176	0.8775
2352	2372	0.8775

3162	3182	0.8775
3160	3180	0.8775
2351	2371	0.8775
3158	3178	0.8775
1659	1679	0.8774
2406	2426	0.8772
2033	2053	0.8772
2405	2425	0.8771
2404	2424	0.8771
1015	1035	0.8771
2408	2428	0.8769
777	797	0.8767
534	554	0.8765
1512	1532	0.8757
1513	1533	0.8757
1510	1530	0.8757
1511	1531	0.8757
794	814	0.8756
1514	1534	0.8756
374	394	0.8755
793	813	0.8755
792	812	0.8755
1570	1590	0.8753
293	313	0.8753
292	312	0.8753
290	310	0.8753
1567	1587	0.8753
289	309	0.8753
291	311	0.8753
952	972	0.8752
774	794	0.8752
339	359	0.8752
294	314	0.8752
775	795	0.8752
1624	1644	0.8751
47	67	0.8751
1067	1087	0.8749
1362	1382	0.8749
825	845	0.8749
1950	1970	0.8749
2933	2953	0.8748
2931	2951	0.8748
2627	2647	0.8748
45	65	0.8748
2932	2952	0.8748
2298	2318	0.8748
2623	2643	0.8747
2622	2642	0.8747
1983	2003	0.8747
1923	1943	0.8746
695	715	0.8746
1618	1638	0.8746
2621	2641	0.8746
798	818	0.8744
800	820	0.8744
2195	2215	0.8744

801	821	0.8744
799	819	0.8744
190	210	0.8743
1021	1041	0.8743
195	215	0.8743
828	848	0.8743
1672	1692	0.8743
192	212	0.8743
1673	1693	0.8743
191	211	0.8743
773	793	0.8742
2608	2628	0.8742
772	792	0.8742
44	64	0.8740
2339	2359	0.8739
2340	2360	0.8739
2218	2238	0.8737
1928	1948	0.8736
697	717	0.8736
698	718	0.8736
2286	2306	0.8736
2311	2331	0.8735
2323	2343	0.8734
2322	2342	0.8734
2306	2326	0.8734
2324	2344	0.8734
2556	2576	0.8732
177	197	0.8731
3164	3184	0.8731
3165	3185	0.8731
3163	3183	0.8731
53	73	0.8727
558	578	0.8726
184	204	0.8726
54	74	0.8726
3193	3213	0.8726
559	579	0.8726
52	72	0.8726
185	205	0.8726
1010	1030	0.8725
1787	1807	0.8724
1789	1809	0.8724
1788	1808	0.8724
2437	2457	0.8722
12	32	0.8722
668	688	0.8722
2438	2458	0.8722
1008	1028	0.8721
665	685	0.8721
1600	1620	0.8721
1599	1619	0.8721
1009	1029	0.8721
1354	1374	0.8721
1007	1027	0.8720
1006	1026	0.8720
1601	1621	0.8720

2548	2568	0.8720
2549	2569	0.8720
182	202	0.8717
180	200	0.8717
181	201	0.8717
121	141	0.8715
1615	1635	0.8715
1614	1634	0.8715
1822	1842	0.8712
1799	1819	0.8711
1542	1562	0.8710
1824	1844	0.8710
1818	1838	0.8707
1820	1840	0.8707
1816	1836	0.8707
3054	3074	0.8707
1817	1837	0.8707
1821	1841	0.8707
1815	1835	0.8707
1819	1839	0.8707
1930	1950	0.8706
3052	3072	0.8705
1081	1101	0.8704
3350	3370	0.8704
1079	1099	0.8704
1080	1100	0.8704
1063	1083	0.8703
2845	2865	0.8703
150	170	0.8702
3014	3034	0.8702
1811	1831	0.8701
370	390	0.8701
1352	1372	0.8697
1035	1055	0.8696
1034	1054	0.8696
3365	3385	0.8694
2647	2667	0.8689
2883	2903	0.8688
2881	2901	0.8686
2385	2405	0.8685
2387	2407	0.8684
2386	2406	0.8684
136	156	0.8683
137	157	0.8683
3320	3340	0.8682
3319	3339	0.8682
3321	3341	0.8682
3324	3344	0.8682
3322	3342	0.8682
3323	3343	0.8682
3308	3328	0.8681
3309	3329	0.8681
142	162	0.8680
215	235	0.8679
487	507	0.8679
214	234	0.8679

1617	1637	0.8672
3353	3373	0.8672
3002	3022	0.8672
3352	3372	0.8670
930	950	0.8669
2034	2054	0.8668
1056	1076	0.8667
1057	1077	0.8667
282	302	0.8666
281	301	0.8666
280	300	0.8666
2733	2753	0.8663
2732	2752	0.8663
643	663	0.8657
2737	2757	0.8656
2736	2756	0.8655
2735	2755	0.8655
2734	2754	0.8655
273	293	0.8655
1658	1678	0.8649
1660	1680	0.8649
481	501	0.8649
1657	1677	0.8649
1012	1032	0.8647
1011	1031	0.8647
171	191	0.8645
2409	2429	0.8644
2410	2430	0.8644
2412	2432	0.8644
2078	2098	0.8644
297	317	0.8644
2411	2431	0.8644
2079	2099	0.8643
1018	1038	0.8642
1017	1037	0.8642
776	796	0.8638
58	78	0.8634
535	555	0.8633
373	393	0.8630
372	392	0.8630
1569	1589	0.8628
1568	1588	0.8628
1065	1085	0.8627
1066	1086	0.8627
2493	2513	0.8626
2494	2514	0.8626
1366	1386	0.8625
2790	2810	0.8625
1369	1389	0.8625
2294	2314	0.8624
2297	2317	0.8624
1363	1383	0.8624
2470	2490	0.8624
2295	2315	0.8624
2296	2316	0.8624
1365	1385	0.8624

1361	1381	0.8623
3071	3091	0.8623
966	986	0.8623
1357	1377	0.8623
1359	1379	0.8623
3072	3092	0.8623
1360	1380	0.8623
3070	3090	0.8622
1925	1945	0.8621
1924	1944	0.8621
832	852	0.8621
2424	2444	0.8620
967	987	0.8620
797	817	0.8619
1805	1825	0.8618
193	213	0.8618
194	214	0.8618
2781	2801	0.8617
515	535	0.8617
2782	2802	0.8617
2780	2800	0.8617
2522	2542	0.8615
2521	2541	0.8615
3310	3330	0.8614
1594	1614	0.8612
1593	1613	0.8612
2288	2308	0.8611
2287	2307	0.8611
696	716	0.8611
2315	2335	0.8609
2709	2729	0.8608
2710	2730	0.8608
2554	2574	0.8607
2717	2737	0.8607
2555	2575	0.8607
2316	2336	0.8606
2317	2337	0.8605
2318	2338	0.8605
986	1006	0.8604
2544	2564	0.8603
2890	2910	0.8602
1106	1126	0.8601
1108	1128	0.8601
1107	1127	0.8601
2401	2421	0.8599
2720	2740	0.8599
2889	2909	0.8598
1781	1801	0.8598
667	687	0.8597
666	686	0.8596
1356	1376	0.8596
1355	1375	0.8596
878	898	0.8596
1019	1039	0.8595
2550	2570	0.8595
2551	2571	0.8594

1948	1968	0.8594
1947	1967	0.8594
1949	1969	0.8594
2721	2741	0.8593
1946	1966	0.8593
3104	3124	0.8591
1574	1594	0.8589
3349	3369	0.8589
3348	3368	0.8588
3141	3161	0.8584
3143	3163	0.8584
3142	3162	0.8584
3055	3075	0.8582
3053	3073	0.8581
3009	3029	0.8577
3008	3028	0.8577
3010	3030	0.8577
3012	3032	0.8577
2194	2214	0.8577
3013	3033	0.8577
3011	3031	0.8577
1808	1828	0.8576
1807	1827	0.8576
1809	1829	0.8576
1062	1082	0.8573
3361	3381	0.8572
3362	3382	0.8572
3363	3383	0.8568
2269	2289	0.8568
2397	2417	0.8565
3029	3049	0.8564
135	155	0.8558
3317	3337	0.8557
15	35	0.8557
3318	3338	0.8557
3316	3336	0.8557
1518	1538	0.8556
16	36	0.8554
1042	1062	0.8553
1520	1540	0.8551
1522	1542	0.8551
1521	1541	0.8551
1523	1543	0.8550
1616	1636	0.8547
479	499	0.8546
464	484	0.8546
2543	2563	0.8544
2541	2561	0.8544
2542	2562	0.8544
284	304	0.8543
285	305	0.8543
216	236	0.8542
218	238	0.8542
3354	3374	0.8541
2524	2544	0.8541
275	295	0.8539

2930	2950	0.8538
2731	2751	0.8538
278	298	0.8535
151	171	0.8535
781	801	0.8535
642	662	0.8531
311	331	0.8531
3360	3380	0.8530
2118	2138	0.8530
2119	2139	0.8530
2341	2361	0.8529
641	661	0.8529
272	292	0.8529
2491	2511	0.8528
2342	2362	0.8526
2344	2364	0.8526
482	502	0.8526
2343	2363	0.8526
2350	2370	0.8525
2349	2369	0.8525
2347	2367	0.8525
2345	2365	0.8525
2346	2366	0.8525
2348	2368	0.8525
2407	2427	0.8522
1013	1033	0.8521
2418	2438	0.8521
1014	1034	0.8521
2417	2437	0.8521
2413	2433	0.8519
2415	2435	0.8519
2414	2434	0.8519
2080	2100	0.8518
778	798	0.8517
55	75	0.8513
699	719	0.8510
700	720	0.8510
701	721	0.8510
59	79	0.8509
1509	1529	0.8507
1016	1036	0.8506
795	815	0.8506
1515	1535	0.8505
382	402	0.8503
338	358	0.8502
951	971	0.8502
340	360	0.8502
823	843	0.8500
1367	1387	0.8500
1054	1074	0.8500
1368	1388	0.8500
1055	1075	0.8500
1625	1645	0.8500
1566	1586	0.8499
2299	2319	0.8499
824	844	0.8499

963	983	0.8498
1358	1378	0.8498
2301	2321	0.8498
2303	2323	0.8498
2305	2325	0.8498
2302	2322	0.8498
2304	2324	0.8498
833	853	0.8496
968	988	0.8496
834	854	0.8496
461	481	0.8496
796	816	0.8496
462	482	0.8496
457	477	0.8495
458	478	0.8495
836	856	0.8495
835	855	0.8495
2787	2807	0.8494
1536	1556	0.8494
2612	2632	0.8494
2611	2631	0.8493
456	476	0.8493
514	534	0.8492
41	61	0.8490
2219	2239	0.8490
2523	2543	0.8490
43	63	0.8490
42	62	0.8490
2619	2639	0.8489
2620	2640	0.8489
771	791	0.8486
2670	2690	0.8486
2671	2691	0.8485
2673	2693	0.8485
2672	2692	0.8485
2711	2731	0.8483
1020	1040	0.8483
2553	2573	0.8482
985	1005	0.8480
984	1004	0.8480
2891	2911	0.8479
916	936	0.8479
2499	2519	0.8479
1592	1612	0.8478
1591	1611	0.8478
561	581	0.8477
560	580	0.8477
562	582	0.8477
557	577	0.8475
2674	2694	0.8474
1790	1810	0.8474
1791	1811	0.8474
183	203	0.8473
1571	1591	0.8473
1572	1592	0.8473
11	31	0.8472

2722	2742	0.8469
1944	1964	0.8468
1945	1965	0.8468
120	140	0.8466
119	139	0.8466
1342	1362	0.8465
346	366	0.8464
347	367	0.8462
114	134	0.8461
348	368	0.8461
1543	1563	0.8460
113	133	0.8459
1519	1539	0.8459
920	940	0.8457
1814	1834	0.8457
1931	1951	0.8456
2017	2037	0.8454
2015	2035	0.8454
1064	1084	0.8454
2016	2036	0.8454
1061	1081	0.8452
1813	1833	0.8452
1812	1832	0.8452
1806	1826	0.8451
369	389	0.8451
2878	2898	0.8450
2880	2900	0.8448
3366	3386	0.8444
2396	2416	0.8440
2394	2414	0.8439
2393	2413	0.8439
2395	2415	0.8439
2648	2668	0.8438
2677	2697	0.8436
2676	2696	0.8436
2388	2408	0.8435
134	154	0.8435
2391	2411	0.8435
133	153	0.8435
2392	2412	0.8435
2389	2409	0.8435
2390	2410	0.8435
2685	2705	0.8434
2686	2706	0.8434
138	158	0.8433
139	159	0.8433
1516	1536	0.8431
1517	1537	0.8431
644	664	0.8429
17	37	0.8429
1526	1546	0.8426
1525	1545	0.8426
1524	1544	0.8425
466	486	0.8422
3003	3023	0.8422
469	489	0.8422

2729	2749	0.8421
477	497	0.8421
478	498	0.8421
931	951	0.8421
2540	2560	0.8419
219	239	0.8417
220	240	0.8417
217	237	0.8417
1058	1078	0.8417
283	303	0.8416
3355	3375	0.8416
2531	2551	0.8415
2528	2548	0.8415
2529	2549	0.8415
2530	2550	0.8415
2532	2552	0.8415
279	299	0.8414
227	247	0.8414
2730	2750	0.8414
222	242	0.8414
223	243	0.8413
2725	2745	0.8413
3357	3377	0.8413
2929	2949	0.8411
3358	3378	0.8410
3359	3379	0.8410
2535	2555	0.8409
2538	2558	0.8409
2534	2554	0.8409
2533	2553	0.8408
2539	2559	0.8408
4	24	0.8407
3	23	0.8407
5	25	0.8407
2	22	0.8407
2738	2758	0.8406
2739	2759	0.8406
3106	3126	0.8405
2928	2948	0.8405
2927	2947	0.8405
271	291	0.8404
270	290	0.8404
2131	2151	0.8404
2132	2152	0.8403
3006	3026	0.8403
3007	3027	0.8403
3005	3025	0.8403
3004	3024	0.8403
640	660	0.8402
1656	1676	0.8401
108	128	0.8399
2032	2052	0.8397
2416	2436	0.8396
2081	2101	0.8393
163	183	0.8390
56	76	0.8387

57	77	0.8387
2293	2313	0.8387
3050	3070	0.8387
3051	3071	0.8386
2846	2866	0.8385
664	684	0.8385
536	556	0.8383
61	81	0.8383
60	80	0.8383
3311	3331	0.8380
513	533	0.8378
335	355	0.8377
337	357	0.8377
336	356	0.8377
2495	2515	0.8376
2520	2540	0.8376
1370	1390	0.8375
964	984	0.8373
131	151	0.8373
965	985	0.8373
132	152	0.8373
2300	2320	0.8373
1375	1395	0.8373
1376	1396	0.8373
1377	1397	0.8373
2779	2799	0.8372
2471	2491	0.8372
460	480	0.8371
1926	1946	0.8371
837	857	0.8370
109	129	0.8370
459	479	0.8370
1927	1947	0.8370
2789	2809	0.8369
802	822	0.8369
2788	2808	0.8369
2613	2633	0.8369
2609	2629	0.8368
2610	2630	0.8368
2220	2240	0.8365
2662	2682	0.8364
2617	2637	0.8364
2618	2638	0.8364
1466	1486	0.8364
2661	2681	0.8361
76	96	0.8361
2422	2442	0.8361
2421	2441	0.8361
2291	2311	0.8360
2289	2309	0.8360
2669	2689	0.8360
2290	2310	0.8360
2715	2735	0.8358
2716	2736	0.8358
2712	2732	0.8358
2423	2443	0.8358

2713	2733	0.8358
983	1003	0.8355
170	190	0.8353
1792	1812	0.8350
1793	1813	0.8350
2740	2760	0.8350
2552	2572	0.8350
879	899	0.8349
880	900	0.8349
877	897	0.8348
10	30	0.8347
1474	1494	0.8346
2723	2743	0.8346
1109	1129	0.8345
1794	1814	0.8344
9	29	0.8344
122	142	0.8340
1110	1130	0.8340
1798	1818	0.8339
1341	1361	0.8339
488	508	0.8339
1573	1593	0.8339
118	138	0.8337
112	132	0.8335
115	135	0.8335
727	747	0.8327
2879	2899	0.8323
1576	1596	0.8321
1577	1597	0.8321
3315	3335	0.8320
2116	2136	0.8317
2117	2137	0.8317
3031	3051	0.8314
3030	3050	0.8314
563	583	0.8311
2675	2695	0.8310
2683	2703	0.8307
2684	2704	0.8307
140	160	0.8306
2649	2669	0.8305
971	991	0.8305
969	989	0.8305
141	161	0.8305
970	990	0.8305
18	38	0.8304
20	40	0.8304
19	39	0.8304
2525	2545	0.8298
1043	1063	0.8298
1535	1555	0.8298
471	491	0.8297
468	488	0.8297
467	487	0.8297
1044	1064	0.8297
465	485	0.8297
463	483	0.8296

472	492	0.8296
221	241	0.8295
473	493	0.8295
1059	1079	0.8292
1060	1080	0.8292
2724	2744	0.8289
226	246	0.8289
780	800	0.8289
225	245	0.8289
224	244	0.8287
2193	2213	0.8286
2192	2212	0.8286
152	172	0.8285
2189	2209	0.8285
2727	2747	0.8285
2726	2746	0.8285
2536	2556	0.8284
2537	2557	0.8284
765	785	0.8283
268	288	0.8282
269	289	0.8282
1	21	0.8282
2125	2145	0.8281
2124	2144	0.8281
3105	3125	0.8281
2120	2140	0.8281
2129	2149	0.8281
663	683	0.8280
228	248	0.8279
2130	2150	0.8279
2133	2153	0.8278
72	92	0.8277
2490	2510	0.8276
1655	1675	0.8276
2489	2509	0.8275
312	332	0.8275
1653	1673	0.8274
2030	2050	0.8272
2917	2937	0.8272
2031	2051	0.8272
107	127	0.8272
2419	2439	0.8270
2921	2941	0.8270
2082	2102	0.8268
779	799	0.8267
702	722	0.8260
1507	1527	0.8256
1508	1528	0.8256
67	87	0.8255
950	970	0.8251
2895	2915	0.8251
2268	2288	0.8250
2896	2916	0.8250
1372	1392	0.8250
1374	1394	0.8249
819	839	0.8249

822	842	0.8249
1564	1584	0.8249
820	840	0.8249
68	88	0.8249
130	150	0.8248
1626	1646	0.8248
2778	2798	0.8247
1378	1398	0.8247
1379	1399	0.8247
1380	1400	0.8247
267	287	0.8245
2614	2634	0.8244
2615	2635	0.8244
2616	2636	0.8244
454	474	0.8243
455	475	0.8243
110	130	0.8243
40	60	0.8240
39	59	0.8240
2517	2537	0.8240
1467	1487	0.8237
2664	2684	0.8237
2660	2680	0.8236
2714	2734	0.8233
2916	2936	0.8232
918	938	0.8232
917	937	0.8232
2915	2935	0.8231
2893	2913	0.8230
2914	2934	0.8230
2892	2912	0.8230
2498	2518	0.8229
914	934	0.8228
915	935	0.8228
2668	2688	0.8228
874	894	0.8227
875	895	0.8227
876	896	0.8227
169	189	0.8225
645	665	0.8224
1469	1489	0.8222
1943	1963	0.8219
3140	3160	0.8218
1472	1492	0.8218
345	365	0.8214
919	939	0.8212
1575	1595	0.8212
116	136	0.8212
117	137	0.8212
349	369	0.8211
1545	1565	0.8210
1544	1564	0.8210
1933	1953	0.8208
1932	1952	0.8208
3368	3388	0.8205
730	750	0.8203

729	749	0.8203
368	388	0.8201
1588	1608	0.8199
1589	1609	0.8199
1590	1610	0.8198
1578	1598	0.8196
3313	3333	0.8195
3312	3332	0.8195
3314	3334	0.8195
770	790	0.8187
1383	1403	0.8187
769	789	0.8187
768	788	0.8187
2678	2698	0.8186
2679	2699	0.8186
564	584	0.8185
2681	2701	0.8181
2680	2700	0.8181
2020	2040	0.8176
2018	2038	0.8176
1527	1547	0.8176
2021	2041	0.8176
2019	2039	0.8176
1528	1548	0.8176
342	362	0.8174
341	361	0.8172
470	490	0.8172
932	952	0.8172
475	495	0.8171
474	494	0.8171
476	496	0.8171
760	780	0.8168
764	784	0.8167
3356	3376	0.8163
2188	2208	0.8161
2728	2748	0.8160
2190	2210	0.8160
0	20	0.8157
2126	2146	0.8156
2123	2143	0.8156
662	682	0.8155
2115	2135	0.8153
2926	2946	0.8152
1385	1405	0.8152
2925	2945	0.8152
2488	2508	0.8152
73	93	0.8151
2924	2944	0.8151
352	372	0.8150
106	126	0.8149
1652	1672	0.8149
2026	2046	0.8148
2028	2048	0.8148
74	94	0.8148
2027	2047	0.8148
2029	2049	0.8147

2918	2938	0.8146
2919	2939	0.8146
2420	2440	0.8145
2920	2940	0.8145
2085	2105	0.8144
2086	2106	0.8144
2083	2103	0.8143
2084	2104	0.8143
62	82	0.8140
162	182	0.8140
63	83	0.8140
64	84	0.8140
161	181	0.8140
160	180	0.8139
164	184	0.8138
165	185	0.8138
3049	3069	0.8138
1053	1073	0.8135
2518	2538	0.8135
383	403	0.8132
6	26	0.8130
317	337	0.8128
2496	2516	0.8126
334	354	0.8126
1371	1391	0.8125
1565	1585	0.8124
1563	1583	0.8124
333	353	0.8124
510	530	0.8123
2847	2867	0.8122
2777	2797	0.8122
2472	2492	0.8122
838	858	0.8122
2848	2868	0.8122
2221	2241	0.8121
1381	1401	0.8121
556	576	0.8121
129	149	0.8121
266	286	0.8120
78	98	0.8120
512	532	0.8119
2134	2154	0.8119
2135	2155	0.8119
1382	1402	0.8119
111	131	0.8118
803	823	0.8118
804	824	0.8118
77	97	0.8117
37	57	0.8115
2653	2673	0.8114
2516	2536	0.8114
2663	2683	0.8112
1468	1488	0.8112
1465	1485	0.8112
35	55	0.8111
75	95	0.8111

80	100	0.8111
2292	2312	0.8111
79	99	0.8111
2665	2685	0.8110
2666	2686	0.8110
2658	2678	0.8107
2659	2679	0.8107
982	1002	0.8106
2742	2762	0.8106
981	1001	0.8105
2744	2764	0.8105
2913	2933	0.8105
2912	2932	0.8105
2500	2520	0.8104
2655	2675	0.8103
2654	2674	0.8103
2656	2676	0.8102
2657	2677	0.8102
2667	2687	0.8102
1475	1495	0.8101
881	901	0.8100
2025	2045	0.8100
646	666	0.8099
882	902	0.8099
647	667	0.8098
2873	2893	0.8098
1473	1493	0.8096
906	926	0.8096
1796	1816	0.8094
1797	1817	0.8094
1795	1815	0.8094
2024	2044	0.8092
761	781	0.8091
974	994	0.8090
973	993	0.8089
972	992	0.8089
344	364	0.8089
1340	1360	0.8087
235	255	0.8084
975	995	0.8083
976	996	0.8083
3367	3387	0.8083
731	751	0.8078
728	748	0.8077
638	658	0.8077
726	746	0.8077
2877	2897	0.8076
734	754	0.8075
1586	1606	0.8074
1585	1605	0.8074
30	50	0.8074
1579	1599	0.8073
1580	1600	0.8073
1581	1601	0.8073
639	659	0.8070
2909	2929	0.8067

759	779	0.8065
767	787	0.8063
766	786	0.8063
566	586	0.8060
2682	2702	0.8056
22	42	0.8054
23	43	0.8054
21	41	0.8054
2023	2043	0.8051
2022	2042	0.8051
1529	1549	0.8050
2526	2546	0.8048
1534	1554	0.8047
2527	2547	0.8040
2191	2211	0.8036
265	285	0.8036
412	432	0.8033
413	433	0.8032
2122	2142	0.8031
2121	2141	0.8031
2128	2148	0.8031
3107	3127	0.8031
71	91	0.8028
1387	1407	0.8027
1654	1674	0.8026
351	371	0.8025
2477	2497	0.8024
2474	2494	0.8024
2473	2493	0.8024
2476	2496	0.8024
2475	2495	0.8024
229	249	0.8024
2923	2943	0.8023
313	333	0.8022
104	124	0.8021
314	334	0.8021
2922	2942	0.8020
2114	2134	0.8019
166	186	0.8018
712	732	0.8013
713	733	0.8013
155	175	0.8011
159	179	0.8011
158	178	0.8011
2519	2539	0.8011
704	724	0.8010
703	723	0.8010
65	85	0.8009
1506	1526	0.8008
153	173	0.8008
537	557	0.8008
154	174	0.8008
8	28	0.8006
66	86	0.8006
167	187	0.8005
168	188	0.8005

705	725	0.8003
318	338	0.8003
649	669	0.8000
1046	1066	0.8000
1045	1065	0.7999
949	969	0.7999
818	838	0.7999
69	89	0.7999
816	836	0.7999
817	837	0.7999
1373	1393	0.7999
821	841	0.7999
511	531	0.7998
1562	1582	0.7998
1627	1647	0.7997
1391	1411	0.7997
3032	3052	0.7995
659	679	0.7994
38	58	0.7990
36	56	0.7990
2652	2672	0.7990
2650	2670	0.7990
409	429	0.7987
1111	1131	0.7984
2894	2914	0.7981
980	1000	0.7981
2741	2761	0.7981
885	905	0.7981
2743	2763	0.7980
1477	1497	0.7977
873	893	0.7977
236	256	0.7974
648	668	0.7973
2874	2894	0.7972
1470	1490	0.7969
1471	1491	0.7968
763	783	0.7967
762	782	0.7967
124	144	0.7965
491	511	0.7965
123	143	0.7965
489	509	0.7964
126	146	0.7964
125	145	0.7964
343	363	0.7964
490	510	0.7964
2899	2919	0.7963
2900	2920	0.7963
350	370	0.7963
978	998	0.7961
1553	1573	0.7961
977	997	0.7961
2897	2917	0.7960
1552	1572	0.7959
234	254	0.7959
1934	1954	0.7958

1936	1956	0.7958
1935	1955	0.7958
3369	3389	0.7955
910	930	0.7953
27	47	0.7952
28	48	0.7952
29	49	0.7952
725	745	0.7951
945	965	0.7951
634	654	0.7951
736	756	0.7950
735	755	0.7950
367	387	0.7950
911	931	0.7950
637	657	0.7950
1584	1604	0.7949
364	384	0.7949
365	385	0.7949
912	932	0.7949
363	383	0.7949
913	933	0.7949
31	51	0.7949
366	386	0.7949
1587	1607	0.7949
580	600	0.7949
907	927	0.7947
909	929	0.7947
2910	2930	0.7941
758	778	0.7940
944	964	0.7940
1496	1516	0.7940
1384	1404	0.7936
565	585	0.7935
567	587	0.7935
568	588	0.7934
406	426	0.7933
1531	1551	0.7924
1532	1552	0.7924
1530	1550	0.7924
1533	1553	0.7922
263	283	0.7915
661	681	0.7911
660	680	0.7911
2186	2206	0.7911
2187	2207	0.7910
942	962	0.7910
941	961	0.7910
939	959	0.7908
940	960	0.7908
2127	2147	0.7906
1386	1406	0.7902
2487	2507	0.7902
2486	2506	0.7902
81	101	0.7902
1114	1134	0.7900
1113	1133	0.7900

353	373	0.7900
1115	1135	0.7900
105	125	0.7897
716	736	0.7896
717	737	0.7895
316	336	0.7895
2087	2107	0.7893
1388	1408	0.7893
933	953	0.7892
935	955	0.7892
934	954	0.7892
938	958	0.7889
937	957	0.7889
715	735	0.7886
157	177	0.7886
156	176	0.7886
384	404	0.7882
1051	1071	0.7882
1052	1072	0.7882
842	862	0.7881
1047	1067	0.7881
1050	1070	0.7880
7	27	0.7879
2267	2287	0.7875
2497	2517	0.7875
812	832	0.7874
813	833	0.7874
453	473	0.7874
814	834	0.7874
815	835	0.7874
545	565	0.7873
1393	1413	0.7873
323	343	0.7873
509	529	0.7873
2776	2796	0.7873
2089	2109	0.7872
1392	1412	0.7872
332	352	0.7871
2137	2157	0.7871
555	575	0.7871
2136	2156	0.7870
3033	3053	0.7869
719	739	0.7868
805	825	0.7868
720	740	0.7868
552	572	0.7865
2651	2671	0.7865
2515	2535	0.7864
886	906	0.7859
658	678	0.7856
657	677	0.7856
884	904	0.7855
2911	2931	0.7855
1481	1501	0.7853
1479	1499	0.7853
1480	1500	0.7853

883	903	0.7853
1476	1496	0.7852
905	925	0.7849
904	924	0.7849
2870	2890	0.7848
239	259	0.7848
2871	2891	0.7846
2872	2892	0.7845
1942	1962	0.7844
3139	3159	0.7843
128	148	0.7842
127	147	0.7841
3370	3390	0.7840
492	512	0.7840
1941	1961	0.7839
2901	2921	0.7837
2902	2922	0.7837
1338	1358	0.7837
1939	1959	0.7837
1938	1958	0.7837
1940	1960	0.7836
1546	1566	0.7836
2898	2918	0.7835
2903	2923	0.7834
1550	1570	0.7834
1551	1571	0.7834
2113	2133	0.7833
1549	1569	0.7833
1547	1567	0.7832
2159	2179	0.7831
732	752	0.7828
733	753	0.7826
722	742	0.7826
635	655	0.7826
26	46	0.7826
723	743	0.7826
2906	2926	0.7825
636	656	0.7824
1582	1602	0.7824
578	598	0.7824
579	599	0.7824
577	597	0.7824
2239	2259	0.7823
2908	2928	0.7823
32	52	0.7823
33	53	0.7822
34	54	0.7822
1192	1212	0.7822
943	963	0.7815
1495	1515	0.7812
414	434	0.7812
2849	2869	0.7810
405	425	0.7805
404	424	0.7805
24	44	0.7804
575	595	0.7803

574	594	0.7803
576	596	0.7803
407	427	0.7803
589	609	0.7788
264	284	0.7788
3108	3128	0.7782
1116	1136	0.7777
2485	2505	0.7777
588	608	0.7776
70	90	0.7776
82	102	0.7775
230	250	0.7774
231	251	0.7774
1651	1671	0.7774
1112	1132	0.7772
233	253	0.7772
315	335	0.7770
2088	2108	0.7768
3048	3068	0.7768
936	956	0.7767
411	431	0.7765
710	730	0.7762
711	731	0.7762
714	734	0.7761
1504	1524	0.7760
1505	1525	0.7759
539	559	0.7757
538	558	0.7757
841	861	0.7756
542	562	0.7756
543	563	0.7756
544	564	0.7756
843	863	0.7755
3047	3067	0.7754
320	340	0.7753
319	339	0.7753
706	726	0.7752
2222	2242	0.7751
321	341	0.7750
2746	2766	0.7750
633	653	0.7750
1464	1484	0.7749
508	528	0.7748
546	566	0.7748
324	344	0.7748
322	342	0.7748
593	613	0.7747
1389	1409	0.7747
839	859	0.7747
1390	1410	0.7747
888	908	0.7746
2769	2789	0.7746
2745	2765	0.7745
1628	1648	0.7745
594	614	0.7745
887	907	0.7745

3035	3055	0.7743
806	826	0.7743
3034	3054	0.7743
2502	2522	0.7741
2501	2521	0.7741
2503	2523	0.7741
554	574	0.7740
331	351	0.7740
2513	2533	0.7740
650	670	0.7739
651	671	0.7739
410	430	0.7737
656	676	0.7734
655	675	0.7732
327	347	0.7732
326	346	0.7732
330	350	0.7731
1483	1503	0.7728
1478	1498	0.7727
237	257	0.7725
241	261	0.7724
238	258	0.7723
240	260	0.7723
415	435	0.7719
1339	1359	0.7712
1555	1575	0.7711
1554	1574	0.7711
738	758	0.7709
743	763	0.7708
1937	1957	0.7707
1548	1568	0.7707
2904	2924	0.7706
2160	2180	0.7706
2240	2260	0.7705
2905	2925	0.7705
3138	3158	0.7704
724	744	0.7701
2875	2895	0.7701
721	741	0.7701
103	123	0.7701
737	757	0.7701
2876	2896	0.7701
756	776	0.7700
2241	2261	0.7699
1583	1603	0.7699
946	966	0.7699
581	601	0.7699
757	777	0.7698
908	928	0.7697
2242	2262	0.7695
1493	1513	0.7686
571	591	0.7685
1191	1211	0.7683
1190	1210	0.7683
1189	1209	0.7682
570	590	0.7682

408	428	0.7682
573	593	0.7678
25	45	0.7677
262	282	0.7664
2185	2205	0.7661
354	374	0.7650
1650	1670	0.7649
2478	2498	0.7649
232	252	0.7647
628	648	0.7646
718	738	0.7645
709	729	0.7638
3046	3066	0.7636
2090	2110	0.7636
847	867	0.7633
844	864	0.7632
385	405	0.7632
540	560	0.7631
541	561	0.7631
1049	1069	0.7630
1048	1068	0.7630
708	728	0.7628
2142	2162	0.7627
1337	1357	0.7627
811	831	0.7627
2141	2161	0.7627
2748	2768	0.7626
2747	2767	0.7625
632	652	0.7625
1395	1415	0.7624
948	968	0.7624
1561	1581	0.7623
596	616	0.7623
595	615	0.7623
1394	1414	0.7623
1462	1482	0.7623
451	471	0.7623
325	345	0.7623
547	567	0.7623
452	472	0.7623
592	612	0.7622
840	860	0.7622
2773	2793	0.7622
2770	2790	0.7622
2775	2795	0.7622
2774	2794	0.7622
631	651	0.7622
2768	2788	0.7621
893	913	0.7619
892	912	0.7619
2511	2531	0.7616
2510	2530	0.7616
2512	2532	0.7616
553	573	0.7615
2266	2286	0.7615
2514	2534	0.7614

361	381	0.7614
362	382	0.7614
652	672	0.7612
3372	3392	0.7609
3373	3393	0.7609
329	349	0.7607
871	891	0.7606
979	999	0.7606
1207	1227	0.7603
1482	1502	0.7603
2869	2889	0.7602
872	892	0.7602
900	920	0.7600
903	923	0.7599
2107	2127	0.7598
2108	2128	0.7598
899	919	0.7594
494	514	0.7592
1560	1580	0.7590
739	759	0.7587
2112	2132	0.7583
2158	2178	0.7583
2110	2130	0.7583
493	513	0.7581
2161	2181	0.7580
2235	2255	0.7580
3371	3391	0.7576
755	775	0.7575
1193	1213	0.7572
2907	2927	0.7572
1503	1523	0.7570
1502	1522	0.7570
1184	1204	0.7569
1195	1215	0.7565
1498	1518	0.7565
1497	1517	0.7565
1494	1514	0.7562
1491	1511	0.7561
2850	2870	0.7560
2852	2872	0.7560
2851	2871	0.7560
569	589	0.7559
403	423	0.7555
590	610	0.7540
1484	1504	0.7538
1485	1505	0.7538
1499	1519	0.7536
3109	3129	0.7532
450	470	0.7530
2484	2504	0.7527
1118	1138	0.7527
1117	1137	0.7527
587	607	0.7526
2482	2502	0.7526
2483	2503	0.7526
749	769	0.7516

750	770	0.7516
748	768	0.7516
1120	1140	0.7515
744	764	0.7508
386	406	0.7504
387	407	0.7504
2140	2160	0.7502
707	727	0.7502
808	828	0.7502
2749	2769	0.7501
2138	2158	0.7501
947	967	0.7499
2184	2204	0.7499
1463	1483	0.7498
597	617	0.7498
807	827	0.7497
551	571	0.7497
889	909	0.7496
1629	1649	0.7495
2243	2263	0.7495
630	650	0.7494
629	649	0.7494
2504	2524	0.7493
2507	2527	0.7493
2505	2525	0.7493
2506	2526	0.7493
3374	3394	0.7492
3036	3056	0.7492
3375	3395	0.7491
890	910	0.7490
891	911	0.7490
653	673	0.7486
848	868	0.7482
328	348	0.7482
654	674	0.7482
901	921	0.7477
1205	1225	0.7476
416	436	0.7469
1558	1578	0.7467
495	515	0.7467
1556	1576	0.7467
1557	1577	0.7467
1559	1579	0.7465
740	760	0.7462
2157	2177	0.7462
741	761	0.7459
2111	2131	0.7458
742	762	0.7458
2237	2257	0.7457
2236	2256	0.7457
2163	2183	0.7457
2162	2182	0.7455
1194	1214	0.7454
2164	2184	0.7452
3137	3157	0.7452
2223	2243	0.7451

2238	2258	0.7450
582	602	0.7449
2230	2250	0.7447
1500	1520	0.7445
1185	1205	0.7443
1186	1206	0.7441
1187	1207	0.7441
1197	1217	0.7441
1188	1208	0.7440
1183	1203	0.7438
1492	1512	0.7436
1490	1510	0.7435
1489	1509	0.7432
572	592	0.7428
1488	1508	0.7418
507	527	0.7407
585	605	0.7401
586	606	0.7401
2480	2500	0.7401
2481	2501	0.7401
584	604	0.7401
355	375	0.7400
2479	2499	0.7400
83	103	0.7400
1649	1669	0.7399
627	647	0.7395
394	414	0.7391
747	767	0.7391
395	415	0.7391
746	766	0.7391
1121	1141	0.7390
2091	2111	0.7386
2092	2112	0.7386
846	866	0.7384
845	865	0.7382
2751	2771	0.7377
810	830	0.7377
809	829	0.7377
2752	2772	0.7377
2750	2770	0.7376
1398	1418	0.7375
388	408	0.7375
2146	2166	0.7375
2143	2163	0.7375
2147	2167	0.7375
1397	1417	0.7374
550	570	0.7374
548	568	0.7374
1396	1416	0.7374
549	569	0.7374
1460	1480	0.7373
1461	1481	0.7373
2771	2791	0.7372
1458	1478	0.7371
2767	2787	0.7371
1459	1479	0.7370

3039	3059	0.7369
2509	2529	0.7367
583	603	0.7366
591	611	0.7365
1214	1234	0.7362
870	890	0.7358
869	889	0.7358
242	262	0.7355
1206	1226	0.7353
3045	3065	0.7350
2109	2129	0.7348
2103	2123	0.7347
2106	2126	0.7347
2102	2122	0.7347
751	771	0.7342
895	915	0.7340
2155	2175	0.7340
2154	2174	0.7340
894	914	0.7340
2156	2176	0.7339
753	773	0.7334
752	772	0.7332
2234	2254	0.7330
102	122	0.7326
754	774	0.7325
2227	2247	0.7323
2226	2246	0.7323
2224	2244	0.7323
2228	2248	0.7323
2225	2245	0.7323
2229	2249	0.7323
2232	2252	0.7322
2231	2251	0.7322
1501	1521	0.7320
2171	2191	0.7316
1196	1216	0.7315
402	422	0.7309
2854	2874	0.7307
2853	2873	0.7307
2855	2875	0.7307
2856	2876	0.7307
1216	1236	0.7302
1122	1142	0.7293
2868	2888	0.7291
2867	2887	0.7291
258	278	0.7291
257	277	0.7289
261	281	0.7289
1486	1506	0.7288
3110	3130	0.7283
360	380	0.7280
1443	1463	0.7279
1336	1356	0.7279
1441	1461	0.7279
2866	2886	0.7277
506	526	0.7274

449	469	0.7267
745	765	0.7266
393	413	0.7266
1119	1139	0.7265
421	441	0.7265
2139	2159	0.7253
2145	2165	0.7250
2265	2285	0.7250
1399	1419	0.7249
598	618	0.7248
2772	2792	0.7247
1630	1650	0.7246
2766	2786	0.7246
2183	2203	0.7245
3038	3058	0.7244
1215	1235	0.7240
505	525	0.7238
502	522	0.7236
501	521	0.7235
244	264	0.7230
1208	1228	0.7229
1201	1221	0.7229
902	922	0.7228
3043	3063	0.7226
1204	1224	0.7226
3042	3062	0.7226
3044	3064	0.7225
2105	2125	0.7222
2104	2124	0.7222
849	869	0.7220
850	870	0.7220
898	918	0.7219
496	516	0.7217
1456	1476	0.7212
419	439	0.7209
420	440	0.7209
2173	2193	0.7206
2233	2253	0.7205
85	105	0.7203
1199	1219	0.7196
1198	1218	0.7191
3136	3156	0.7179
427	447	0.7178
428	448	0.7178
425	445	0.7174
426	446	0.7173
2165	2185	0.7168
2169	2189	0.7168
2170	2190	0.7168
2167	2187	0.7168
2168	2188	0.7167
259	279	0.7166
1487	1507	0.7166
1448	1468	0.7161
1451	1471	0.7160
1335	1355	0.7154

1440	1460	0.7151
1648	1668	0.7149
400	420	0.7147
626	646	0.7144
448	468	0.7143
396	416	0.7141
398	418	0.7141
397	417	0.7141
2761	2781	0.7140
2762	2782	0.7140
2763	2783	0.7137
2093	2113	0.7136
2754	2774	0.7134
2148	2168	0.7125
2144	2164	0.7125
601	621	0.7123
2244	2264	0.7123
2765	2785	0.7123
1631	1651	0.7121
2181	2201	0.7120
1426	1446	0.7120
2182	2202	0.7120
3037	3057	0.7119
2508	2528	0.7118
1211	1231	0.7117
1210	1230	0.7117
1213	1233	0.7111
1200	1220	0.7106
243	263	0.7105
2152	2172	0.7105
2151	2171	0.7104
2860	2880	0.7103
1203	1223	0.7102
422	442	0.7101
1202	1222	0.7101
443	463	0.7098
2101	2121	0.7097
852	872	0.7097
868	888	0.7096
897	917	0.7094
417	437	0.7094
859	879	0.7094
418	438	0.7093
497	517	0.7092
896	916	0.7090
500	520	0.7089
499	519	0.7089
2153	2173	0.7089
498	518	0.7089
1457	1477	0.7087
861	881	0.7082
84	104	0.7080
1182	1202	0.7066
3040	3060	0.7066
2172	2192	0.7065
3112	3132	0.7063

390	410	0.7061
389	409	0.7061
1217	1237	0.7052
1452	1472	0.7044
1123	1143	0.7043
1124	1144	0.7042
1447	1467	0.7039
260	280	0.7039
2859	2879	0.7035
3111	3131	0.7035
391	411	0.7034
359	379	0.7030
357	377	0.7030
358	378	0.7030
356	376	0.7030
1442	1462	0.7029
1444	1464	0.7028
1334	1354	0.7027
399	419	0.7024
392	412	0.7016
2755	2775	0.7009
2753	2773	0.7002
2150	2170	0.7002
602	622	0.7001
2149	2169	0.7000
599	619	0.6999
1400	1420	0.6999
1425	1445	0.6995
100	120	0.6995
1634	1654	0.6995
1424	1444	0.6994
1209	1229	0.6992
2862	2882	0.6992
1636	1656	0.6992
2863	2883	0.6991
2861	2881	0.6990
97	117	0.6989
504	524	0.6987
503	523	0.6986
245	265	0.6981
851	871	0.6972
2100	2120	0.6972
860	880	0.6968
423	443	0.6968
442	462	0.6965
1455	1475	0.6962
2865	2885	0.6959
90	110	0.6958
863	883	0.6958
862	882	0.6957
865	885	0.6955
101	121	0.6955
86	106	0.6954
3041	3061	0.6954
1181	1201	0.6947
401	421	0.6937

429	449	0.6937
2857	2877	0.6932
441	461	0.6929
1218	1238	0.6928
3135	3155	0.6928
1219	1239	0.6926
424	444	0.6924
430	450	0.6924
252	272	0.6923
254	274	0.6922
253	273	0.6922
2166	2186	0.6918
1453	1473	0.6916
2174	2194	0.6916
256	276	0.6915
1445	1465	0.6915
255	275	0.6914
1450	1470	0.6911
1439	1459	0.6904
440	460	0.6900
438	458	0.6899
439	459	0.6899
1647	1667	0.6897
447	467	0.6893
444	464	0.6892
445	465	0.6892
2764	2784	0.6888
603	623	0.6875
600	620	0.6874
1633	1653	0.6871
1427	1447	0.6870
1430	1450	0.6870
1429	1449	0.6870
99	119	0.6869
2180	2200	0.6869
1212	1232	0.6867
1434	1454	0.6865
98	118	0.6864
1178	1198	0.6862
2094	2114	0.6859
2095	2115	0.6849
855	875	0.6848
856	876	0.6847
2099	2119	0.6847
858	878	0.6847
857	877	0.6847
853	873	0.6847
91	111	0.6833
864	884	0.6831
87	107	0.6830
866	886	0.6830
867	887	0.6821
1437	1457	0.6813
1435	1455	0.6813
1333	1353	0.6801
251	271	0.6793

250	270	0.6793
1125	1145	0.6793
1446	1466	0.6789
1449	1469	0.6786
1438	1458	0.6779
1131	1151	0.6774
437	457	0.6773
625	645	0.6770
446	466	0.6768
2760	2780	0.6765
1220	1240	0.6764
2756	2776	0.6758
1401	1421	0.6750
2264	2284	0.6750
95	115	0.6746
1632	1652	0.6746
1431	1451	0.6746
1635	1655	0.6745
2179	2199	0.6744
2178	2198	0.6744
96	116	0.6744
2177	2197	0.6744
1637	1657	0.6744
2176	2196	0.6743
1423	1443	0.6743
1422	1442	0.6742
2175	2195	0.6742
1433	1453	0.6739
3134	3154	0.6731
2098	2118	0.6720
604	624	0.6716
2864	2884	0.6709
88	108	0.6708
89	109	0.6708
431	451	0.6705
432	452	0.6705
1180	1200	0.6697
3113	3133	0.6687
2096	2116	0.6684
2858	2878	0.6683
2097	2117	0.6682
1332	1352	0.6676
1454	1474	0.6666
249	269	0.6665
1129	1149	0.6664
1128	1148	0.6657
1127	1147	0.6657
1126	1146	0.6657
246	266	0.6650
1646	1666	0.6650
436	456	0.6650
2759	2779	0.6634
2758	2778	0.6634
2245	2265	0.6623
1428	1448	0.6620
92	112	0.6617

1242	1262	0.6617
1243	1263	0.6613
1432	1452	0.6613
1132	1152	0.6611
1133	1153	0.6609
1179	1199	0.6608
1252	1272	0.6603
1251	1271	0.6600
854	874	0.6597
605	625	0.6591
1134	1154	0.6589
1248	1268	0.6584
1247	1267	0.6583
1176	1196	0.6573
1177	1197	0.6572
1436	1456	0.6562
1331	1351	0.6551
624	644	0.6525
2757	2777	0.6509
1402	1422	0.6504
1254	1274	0.6504
611	631	0.6499
1221	1241	0.6499
1416	1436	0.6496
94	114	0.6496
1414	1434	0.6496
612	632	0.6496
1638	1658	0.6495
93	113	0.6494
1421	1441	0.6494
1245	1265	0.6481
1253	1273	0.6478
1137	1157	0.6458
1246	1266	0.6458
433	453	0.6455
1168	1188	0.6454
610	630	0.6452
1169	1189	0.6449
1175	1195	0.6448
1172	1192	0.6443
1174	1194	0.6435
1330	1350	0.6425
1130	1150	0.6420
248	268	0.6409
623	643	0.6402
435	455	0.6400
247	267	0.6400
1645	1665	0.6398
1255	1275	0.6373
1410	1430	0.6373
1413	1433	0.6372
613	633	0.6371
1640	1660	0.6369
1260	1280	0.6364
1244	1264	0.6362
1408	1428	0.6352

1139	1159	0.6345
1642	1662	0.6343
1135	1155	0.6339
1166	1186	0.6335
1249	1269	0.6334
1138	1158	0.6331
1170	1190	0.6323
1173	1193	0.6319
1301	1321	0.6310
1403	1423	0.6309
1404	1424	0.6309
434	454	0.6306
619	639	0.6284
620	640	0.6284
622	642	0.6278
2246	2266	0.6258
1222	1242	0.6255
2263	2283	0.6250
1256	1276	0.6248
1412	1432	0.6248
1409	1429	0.6248
1241	1261	0.6248
1419	1439	0.6247
1420	1440	0.6247
1415	1435	0.6246
1418	1438	0.6246
1417	1437	0.6246
1639	1659	0.6244
3133	3153	0.6231
1250	1270	0.6225
1407	1427	0.6218
1641	1661	0.6218
1406	1426	0.6218
606	626	0.6216
1136	1156	0.6215
1140	1160	0.6210
1287	1307	0.6209
1171	1191	0.6208
1143	1163	0.6206
609	629	0.6202
1167	1187	0.6201
3114	3134	0.6188
614	634	0.6172
615	635	0.6169
616	636	0.6169
618	638	0.6166
621	641	0.6158
1643	1663	0.6148
1644	1664	0.6147
1411	1431	0.6123
1144	1164	0.6121
1259	1279	0.6119
1146	1166	0.6119
1258	1278	0.6119
1299	1319	0.6118
1300	1320	0.6118

1261	1281	0.6114
1297	1317	0.6112
1298	1318	0.6110
1405	1425	0.6093
607	627	0.6090
1142	1162	0.6085
1141	1161	0.6085
1165	1185	0.6083
1288	1308	0.6083
1290	1310	0.6077
1239	1259	0.6069
1329	1349	0.6050
617	637	0.6043
3115	3135	0.6042
1286	1306	0.6015
1156	1176	0.6014
1224	1244	0.6010
1223	1243	0.6009
1147	1167	0.6003
1162	1182	0.5998
1145	1165	0.5998
1257	1277	0.5998
2247	2267	0.5998
1160	1180	0.5992
1159	1179	0.5981
1240	1260	0.5981
1289	1309	0.5956
1291	1311	0.5951
1296	1316	0.5946
1302	1322	0.5945
1238	1258	0.5943
1157	1177	0.5895
1154	1174	0.5889
1155	1175	0.5889
1150	1170	0.5874
1153	1173	0.5868
1161	1181	0.5867
3131	3151	0.5862
3132	3152	0.5856
1262	1282	0.5850
1284	1304	0.5841
608	628	0.5839
1164	1184	0.5833
1237	1257	0.5819
3116	3136	0.5791
1303	1323	0.5784
1274	1294	0.5777
1225	1245	0.5760
1163	1183	0.5754
1149	1169	0.5750
2262	2282	0.5750
1148	1168	0.5750
1152	1172	0.5742
1151	1171	0.5740
1264	1284	0.5734
1158	1178	0.5731

1263	1283	0.5731
1285	1305	0.5720
1283	1303	0.5719
1292	1312	0.5701
1293	1313	0.5699
1295	1315	0.5696
1269	1289	0.5683
1268	1288	0.5681
1273	1293	0.5677
1327	1347	0.5675
1328	1348	0.5675
1272	1292	0.5675
1304	1324	0.5659
1305	1325	0.5655
1275	1295	0.5655
1226	1246	0.5635
1229	1249	0.5634
1233	1253	0.5629
1232	1252	0.5624
1230	1250	0.5624
2248	2268	0.5623
1265	1285	0.5616
3130	3150	0.5612
1266	1286	0.5594
1294	1314	0.5572
1236	1256	0.5569
1271	1291	0.5553
1270	1290	0.5553
1276	1296	0.5530
3117	3137	0.5518
1228	1248	0.5509
1235	1255	0.5506
1231	1251	0.5499
1315	1335	0.5476
1316	1336	0.5476
1313	1333	0.5475
1312	1332	0.5475
1282	1302	0.5475
1309	1329	0.5471
1267	1287	0.5431
1277	1297	0.5405
1306	1326	0.5405
1279	1299	0.5404
1326	1346	0.5397
1227	1247	0.5385
1234	1254	0.5378
2261	2281	0.5376
3129	3149	0.5363
1314	1334	0.5352
1310	1330	0.5350
1311	1331	0.5350
1318	1338	0.5349
1308	1328	0.5346
1317	1337	0.5309
2249	2269	0.5305
1278	1298	0.5286

1280	1300	0.5284
3118	3138	0.5266
3128	3148	0.5238
1325	1345	0.5238
1319	1339	0.5224
1307	1327	0.5220
1281	1301	0.5217
2250	2270	0.5180
3127	3147	0.5143
3119	3139	0.5127
2260	2280	0.5126
1320	1340	0.5099
1322	1342	0.5095
2251	2271	0.5054
1323	1343	0.5005
1324	1344	0.5003
3120	3140	0.5002
2258	2278	0.4986
1321	1341	0.4973
2257	2277	0.4853
2256	2276	0.4853
3123	3143	0.4835
3124	3144	0.4832
3126	3146	0.4768
3125	3145	0.4767
2259	2279	0.4758
2254	2274	0.4727
2253	2273	0.4727
2255	2275	0.4727
2252	2272	0.4726
3122	3142	0.4636
3121	3141	0.4635

Supplementary Figure:

Clustal Omega alignment of most frequent sequences of each virus identified on Weblogos (Figure 2c).

```
DV1         VVVLGSQEGAMHTALTGATEI 21
DV2         VVVLGSQEGAMHTALTGATEI 21
DV3         VVVLGSQEGAMHTALTGATEI 21
DV4         VTVLGSQEGAMHSALAGATEV 21
JEV         VVALGSQEGGLHQALAGAIVV 21
TBEV        VYNLGDQTGVLLKSLAGVPVA 21
WNV         VIALGSQEGALHQALAGAIPV 21
YFV         VLALGNQEGSLKTALTGAMRV 21
ZKV         VVVLGSQEGAVHTALAGALEA 21
            *  **.* * :  :*:*.
```
